# Vertical structure of Caribbean deep-reef fishes from the altiphotic to deep-sea boundary

**DOI:** 10.1038/s41598-024-69774-w

**Published:** 2024-08-22

**Authors:** Juliette Jacquemont, Simon J. Brandl, Emily P. McFarland, Joachim Claudet, Carole C. Baldwin, Jenna Barrett, Luke Tornabene

**Affiliations:** 1 School of Aquatic and Fishery Sciences, University of Washington, 1122 NE Boat St 98195, Seattle, WA USA; 2 Department of Marine Science, The University of Texas at Austin, Marine Science Institute, 750 Channel View Dr, Port Aransas, TX 78373 USA; 3 Scripps Institution of Oceanography, University of California San Diego, 9500 Gilman Drive, La Jolla, CA 92093 USA; 4 National Center for Scientific Research, PSL Université Paris, CRIOBE, CNRS-EPHE-UPVD, Maison de l’Océan, 195 rue Saint-Jacques, 75005 Paris, France; 5 Department of Vertebrate Zoology, National Museum of Natural History, Smithsonian Institution, Washington, DC USA; 6 National Oceanic & Atmospheric Administration SWFSC, 8901 La Jolla Shores Drive, La Jolla, CA 92037 USA

**Keywords:** Community ecology, Biodiversity, Coral reefs

## Abstract

While recent technical breakthroughs have enabled advances in the description of reefs down to 150 m, the structure and depth zonation of deep-reef communities below 150 m remains largely unknown. Here, we present results from over 10 years of deep-reef fish surveys using human-occupied submersibles at four locations across the Caribbean Sea, constituting one of the only continuous reef-fish surveys from 10 to 480 m (1 site) and 40 to 300 m (3 sites). We identify four vertically stratified deep-reef fish communities between 40 and 300 m bordered by an altiphotic (0–10 m) and a deep-sea (300–480 m) community. We found a strong faunal break around 150 m that separates mesophotic and rariphotic zones and secondary breaks at ~ 70 to 90 m and ~ 180 to 200 m subdividing these zones into upper and lower communities. From 300 to 480 m in Roatán, we found a single fish community dominated by deep-sea families, indicating that the lower boundary of the reef-fish realm occurs at 300 m. No differences were found between communities ranging from 20 to 60 m, suggesting that fishes from the lower altiphotic and upper mesophotic form an ecological continuum. While some variability was observed across sites, the overall depth zonation and key species characterizing depth zones were consistent. Most deep-reef species observed were depth specialists restricted to a single depth zone, but many shallow-reef species extended down to mesophotic depths. Depth segregation among species of a genus was found across ten reef-fish genera and likely constitutes one of the mechanisms driving community distinctiveness and thereby fish diversity across depths.

## Main text

Tropical and temperate reefs are amongst the best described marine ecosystems, yet their study has largely been restricted to shallow depths down to 40 m, corresponding to the traditional limits of non-technical SCUBA diving, depicting a very skewed representation of these ecosystems^1,2^. The recent widespread adoption of closed-circuit rebreather diving coupled with the increased use of remotely operated vehicles (ROVs), baited remote underwater videos, and human-occupied submersibles has enabled a surge in the description of reefs below 40 m, highlighting their diversity and taxonomic distinctiveness with respect to their shallow counterparts^3–5^. Despite these advances, our understanding of the ecology of mesophotic (40–150 m), and even more so of rariphotic (150–300 m) reef communities lags behind that of their shallow counterparts.

Most tropical reef communities surveyed across the world display decreases in fish abundance, biomass and richness with depth^6–8^. While many shallow species have wide depth ranges that extend down to the mesophotic^9^, deep-reef fish species (below 40 m) tend to be depth-endemic and therefore are not found on shallow reefs^6,10,11^. The number of distinct fish communities occurring along the reef slope, as well as the depths of associated faunal breaks, varies regionally^12^ depending on temperature, light availability, and other environmental factors^13–15^. Similarly, the composition of deep-reef fish assemblages at a given depth is known to vary within and between regions^16,17^. Regional gradients of reef-fish richness have been found to dampen with depth, likely a result of increased environmental constraints which lead to convergent filtering of species composition^16^.

Despite recent advances, several data gaps still hamper our understanding of deep-reef fish community structure and its regional variability. First, the limited number of deep-reef survey locations constitutes an important barrier to evaluating global patterns of deep-reef fish diversity and levels of deep-reef fish endemism^16,18^. The limited number of study sites also restricts regional comparisons, which are essential for identifying environmental drivers of deep-reef fish diversity patterns and, in turn, increase our capacity to predict the spatial distribution and composition of deep-reef communities^19,20^. Such information is necessary to elucidate evolutionary and demographic processes at play on deep reefs and to adequately place and design conservation tools that protect the full range of reef diversity^21^. Second, technical limitations of rebreather diving and the scarcity and cost of submersibles and ROVs have restricted most deep-reef fish studies to the upper range of deep reefs (40–120 m)^5^; leading to the continued discovery of new species below those depths^2,10,22^ even in the most heavily sampled sites^23^. While submersible research at several locations in the tropical western Atlantic has established that deep-reef fishes extend down to at least 300 m ^4,7,10,11^, the lower depth limit of deep-reef fish distribution and their transition to deep-sea fish communities remains largely unknown.

Here, we advance the understanding of deep-reef fish community structure using one of the most extensive datasets on deep-reef fish communities, comprising observations down to 480 m collected by submersible diving at four sites in the Caribbean. Using hierarchical clustering and similarity profile analyses, we first test whether distinct communities occur across depths, identify the depth of faunal breaks, and quantify the relative role of nestedness and turn-over in explaining the dissimilarity between these communities. We then evaluate the relative contribution of reef-associated vs. deep-sea families to fish abundance and richness across depths, and test for depth segregation between closely-related species. Lastly, we perform a cross-site similarity analysis to test whether vertical structure and community composition are stable at the regional scale.

## Results

### Abundance and richness across depth

**Figure 1:**
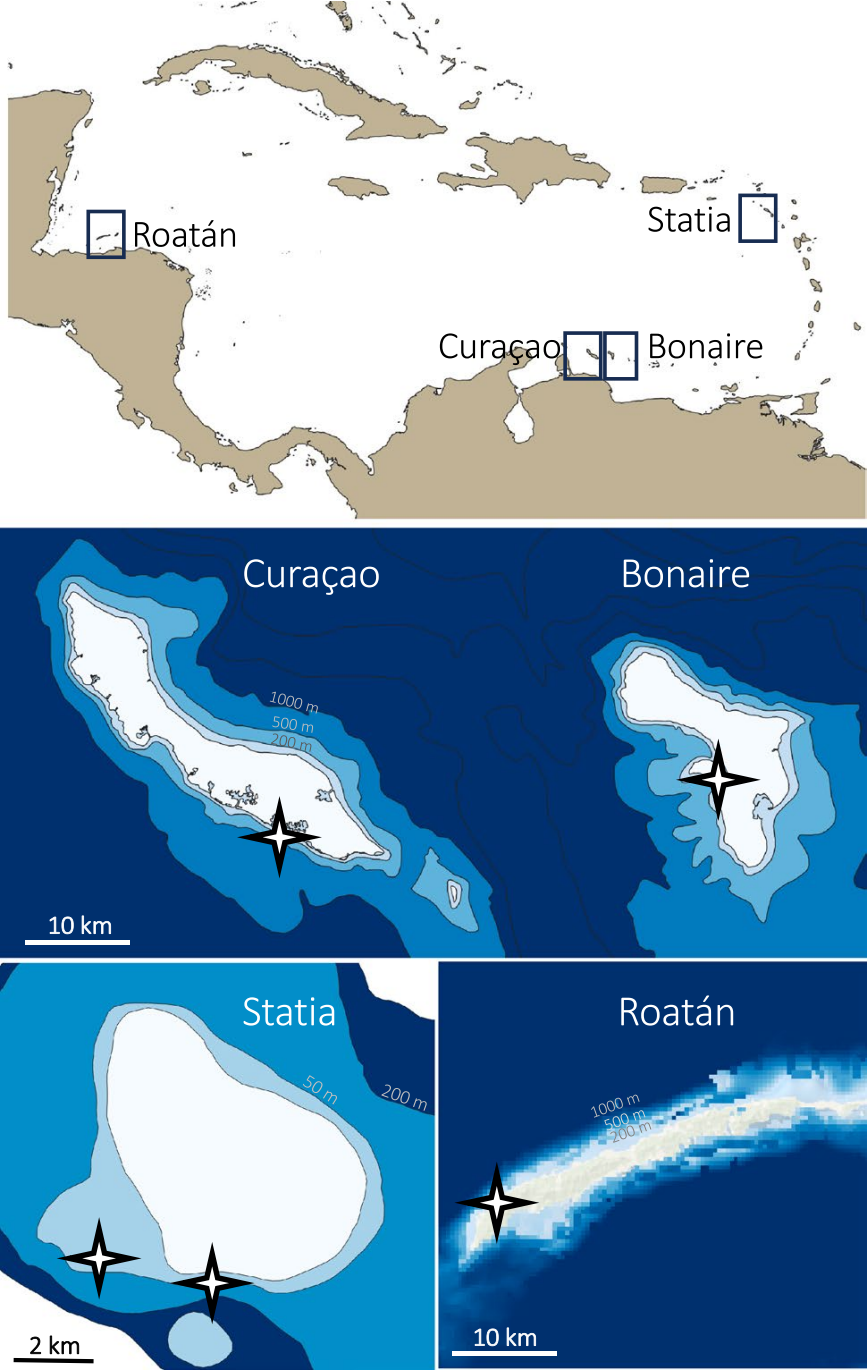
Location of study sites. The bathymetric maps of Curaçao, Bonaire and Statia were obtained from the Dutch Caribbean Biodiversity Database, and the bathymetric map of Roatán from the General Bathymetric Chart of the Ocean.

**Figure 2:**
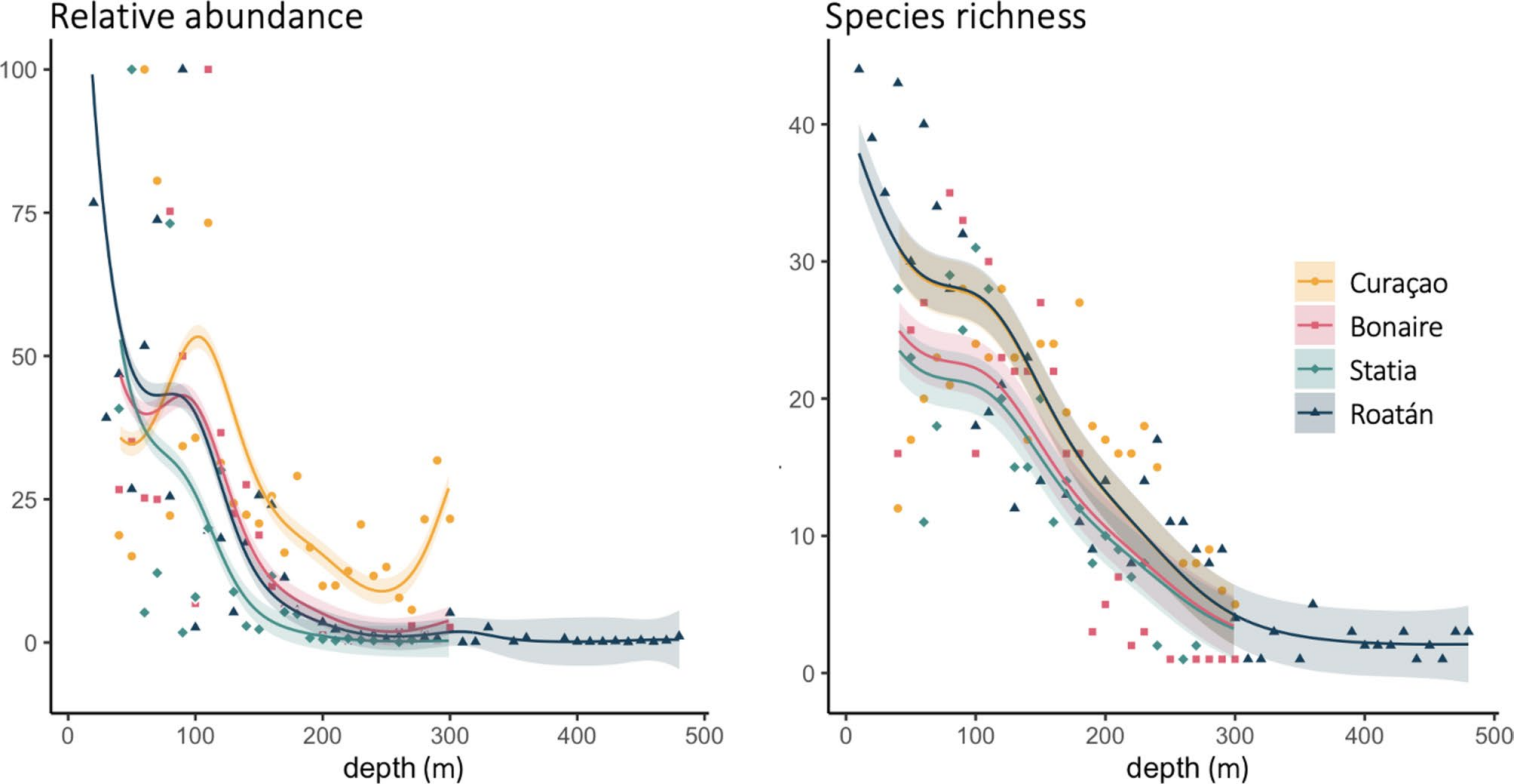
Relative abundance (left) and species richness (right) of fish across depth at the four study sites. Relative abundances were normalized by depth-specific sampling effort and are given as a proportion of the site-specific maximal abundance. Points represent observed relative abundance and richness at each 10 m depth bin, lines represent predicted values from a generalized additive model and shaded areas represent 95% confidence intervals. Note that for depths greater than 300 m and shallower than 40 m, predictions are based on observations from Roatán alone.

A total of 33,873 individuals representing 225 fish species were observed across study sites (Fig. 1) from depths of 10 to 480 m, including 38 deep-reef species that were new to science at the time of their observation (Table S1). Rarefaction curves indicate that sampling effort was sufficient to sample most of the fish diversity in Curaçao, but that additional sampling effort would have likely resulted in additional species observed in Bonaire, Statia, and Roatán (Fig. S1). Fish abundance and species richness decreased with depth at all sites (*p* < 0.001, Fig. 2). Abundance decreased steeply below 80 m depth and plateaued at all sites at 0–10% of initial abundance values below 200 m. The exception was Curaçao, where abundance remained at or above 10% and even increased back to ~ 25% between 270 and 300 m (Fig. 2). Abundance remained stable from 300 to 480 m in Roatán, at 0–3% of initial abundance. Total fish species richness across depth was 73 species in Curaçao, 119 species in Bonaire, 107 species in Statia, and 145 species in Roatán (see Table S1 for complete list of species at each site). At all sites, species richness decreased mostly linearly with depth from 40 to 300 m (Fig. 2). Below 260 m depth, species richness remained below 10 species at all sites, with values ranging between 8 and 10 species in Curacao and Roatán, and 0–2 species in Bonaire and Statia. Species richness halved again below 300 m in Roatán, and plateaued at 2–6 species between 300 and 480 m.

### Vertical structure of fish communities

**Figure 3:**
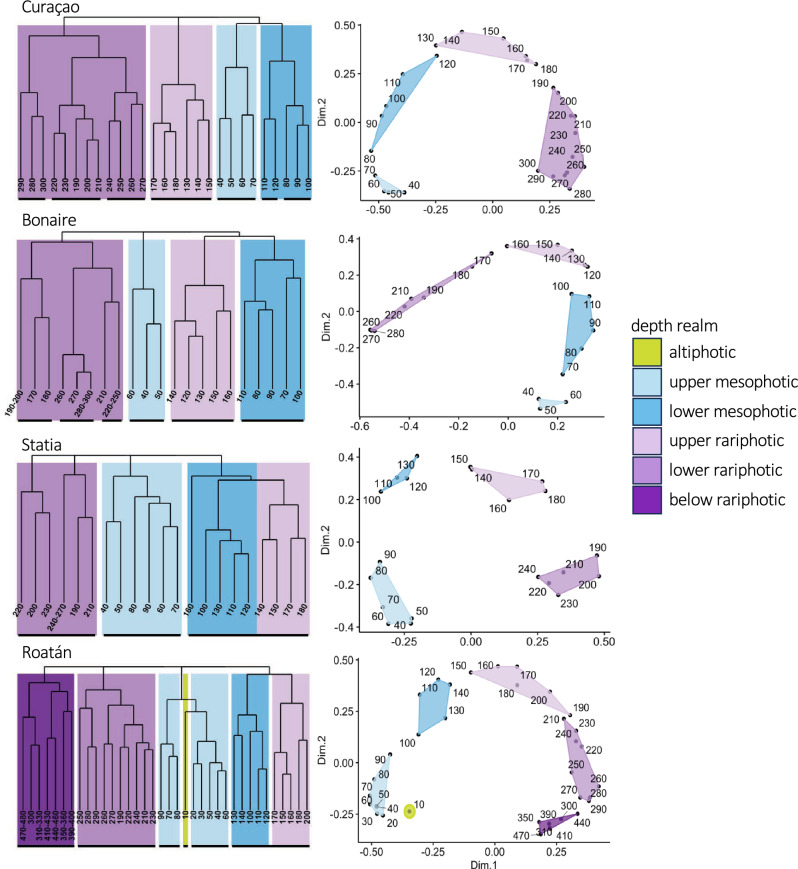
Site-specific dissimilarity analysis of reef-fish community from 40 to 300 m depth. Left panels display hierarchical clustering dendrograms, right panels display nonmetric multidimensional scaling ordination (MDS) plots derived from Bray–Curtis dissimilarity analyses. Clusters with significantly distinct composition (SIMPROF analyses) are indicated on dendrograms by thick continuous horizontal lines below depth bin values. Color scale indicates the depth zones that clusters obtained from SIMPROF analyses were pooled into. Depth-bins are labeled with the minimum depth of each 10 m depth interval (e.g., “100 m” = 100–109 m).

Similarity profile (SIMPROF) analyses based on species composition and abundance at each 10 m depth bin revealed between three (Statia) and seven (Curaçao) distinct fish community clusters between 40 and 300 m (denoted by black thick horizontal lines in Fig. 3). With the exception of the 190 m community in Roatán, which pooled with the 210–300 m communities, all clusters comprised communities from continuously ordered depth bins. Based on main branching events of hierarchical clustering dendrograms (Fig. 3) and on previous analyses of reef-fish depth zonation (Baldwin et al., 2018), SIMPROF clusters were pooled into four depth zones: upper and lower mesophotic, and upper and lower rariphotic. Depth zones formed a posteriori by pooling SIMPROF clusters displayed significantly different fish communities (PERMANOVA, *p*-value < 0.001), and explained between 46% (Statia) and 60% (Curaçao) of the total variance between fish communities at a given site (Fig. 3).

The upper mesophotic zone corresponded to a single cluster starting at 40 m and ending at 70–90 m in Bonaire, Curaçao, and Statia. In Roatán, two sister clusters from 20 to 100 m were considered to form the upper mesophotic. The lower mesophotic corresponded to a single cluster in Bonaire and Roatán and to two sister clusters in Curaçao, and spanned from 70–90 to 120–150 m. In Statia, a single large cluster spanned from 100 to 190 m. For comparative purposes, this cluster was divided into a lower mesophotic and upper rariphotic zone, with a depth break at 140 m corresponding to the main branching event within this cluster (Fig. 3). At all other sites, the upper rariphotic corresponded to a single cluster spanning from 120–150 to 170–210 m. Lastly, the lower rariphotic corresponded to a single cluster in Statia and Roatán, and to three adjacent clusters in Curaçao and Bonaire. Community breaks separating depth zones occurred at similar depths in Curaçao and Bonaire: ~ 70 to 80 m, ~ 120 to 130 m, and ~ 170 to 190 m. At Statia and Roatán, community breaks were skewed towards deeper depths at 100 m, ~ 140 to 150 m, and ~ 190 to 210 m. Two additional community breaks were found in Roatán: one separating the shallowest 10 m depth bin from the next altiphotic/upper mesophotic cluster (20–60 m), and one at 300 m separating the lower rariphotic from a deeper fish community that formed a single cluster down to 470 m (Fig. 3).

### Indicator species across depth zones

**Figure 4:**
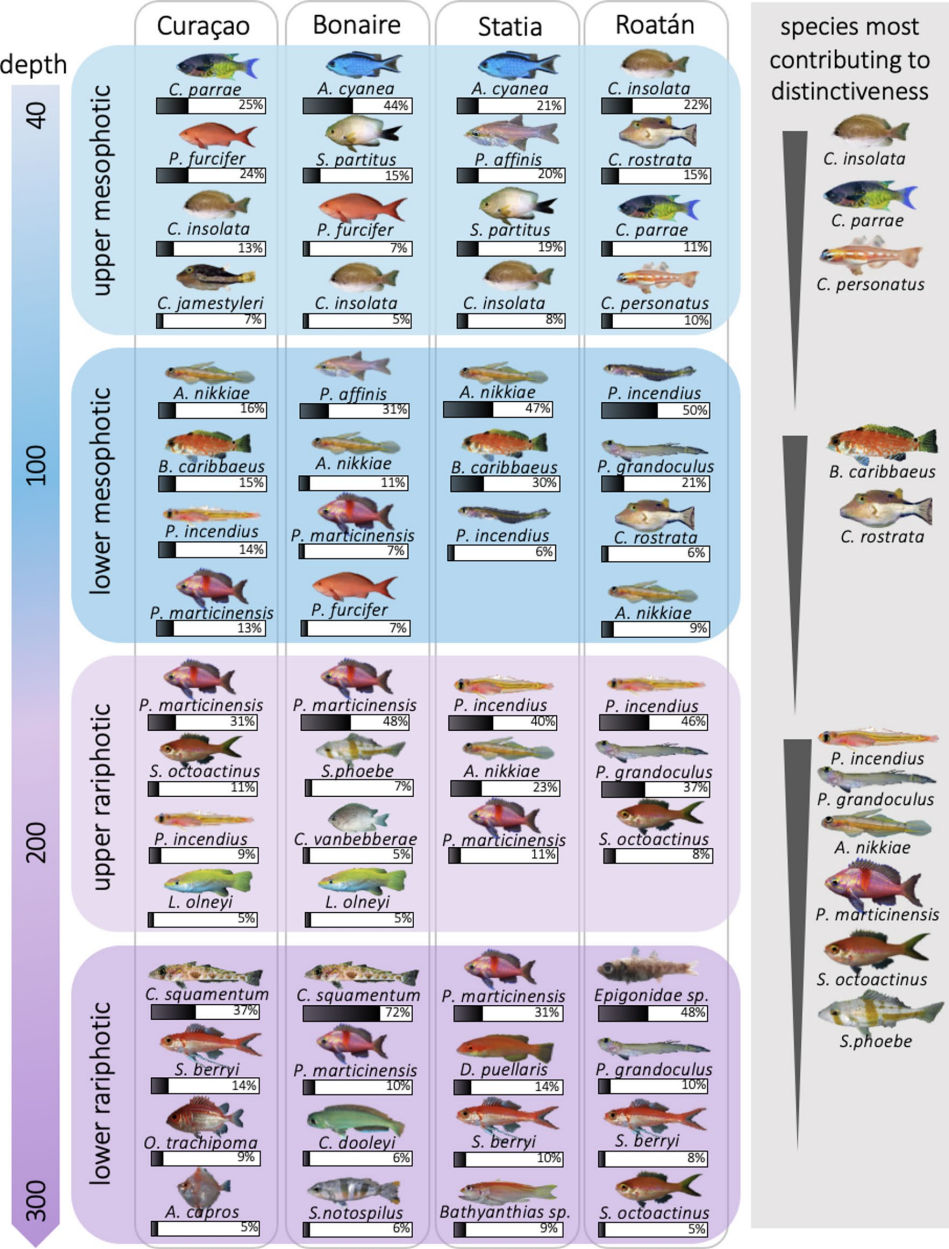
Most common species by depth zone and site. Species displayed are the top four most common species by depth zone and sites, representing at least 5% of the total abundance. The relative abundance of each species per depth zone per site is indicated in %. Species most contributing to differences between depth zones (SIMPER contribution > 5% and *p*-value < 0.05) are displayed in the right grey panel, with abundance of these species being higher in the shallower of the two depth zones.

The top five most abundant fish species per site and per depth zones represented 46–91% of the total community abundance in that depth zone. Most abundant species changed between depth zones but were often identical across sites (Fig. 4). Species that significantly contributed to differences in fish communities between depth zones (SIMPER analyses) were a subset of the most abundant species (Fig. 4). A decrease in the abundance of *Clepticus parrae*, *Chromis insolata,* and *Coryphopterus personatus* marked the transition between the upper and lower mesophotic; a decrease in the abundance of *Canthigaster rostrata* and *Bullisichthys carribaeus* marked the transition between the lower mesophotic and upper rariphotic; and a decrease in the abundance of *Serranus phoebe, Pronotogrammus martinicensis, Symphysanodon octoactinus, Palatogobius incendius, Palatogobius grandoculus,* and *Antilligobius nikkiae* marked the transition between the upper and lower rariphotic. In Roatán, an increase in the abundance of *Hollardia hollardi*, *Epigonidae sp.*, *Chrionema squamentum*, *Phenascoscorpius nebrius*, and *Neoepinnula americana* marked the transition between the lower rariphotic and the next deeper community.

### *Beta*-diversity across depth zones

**Figure 5:**
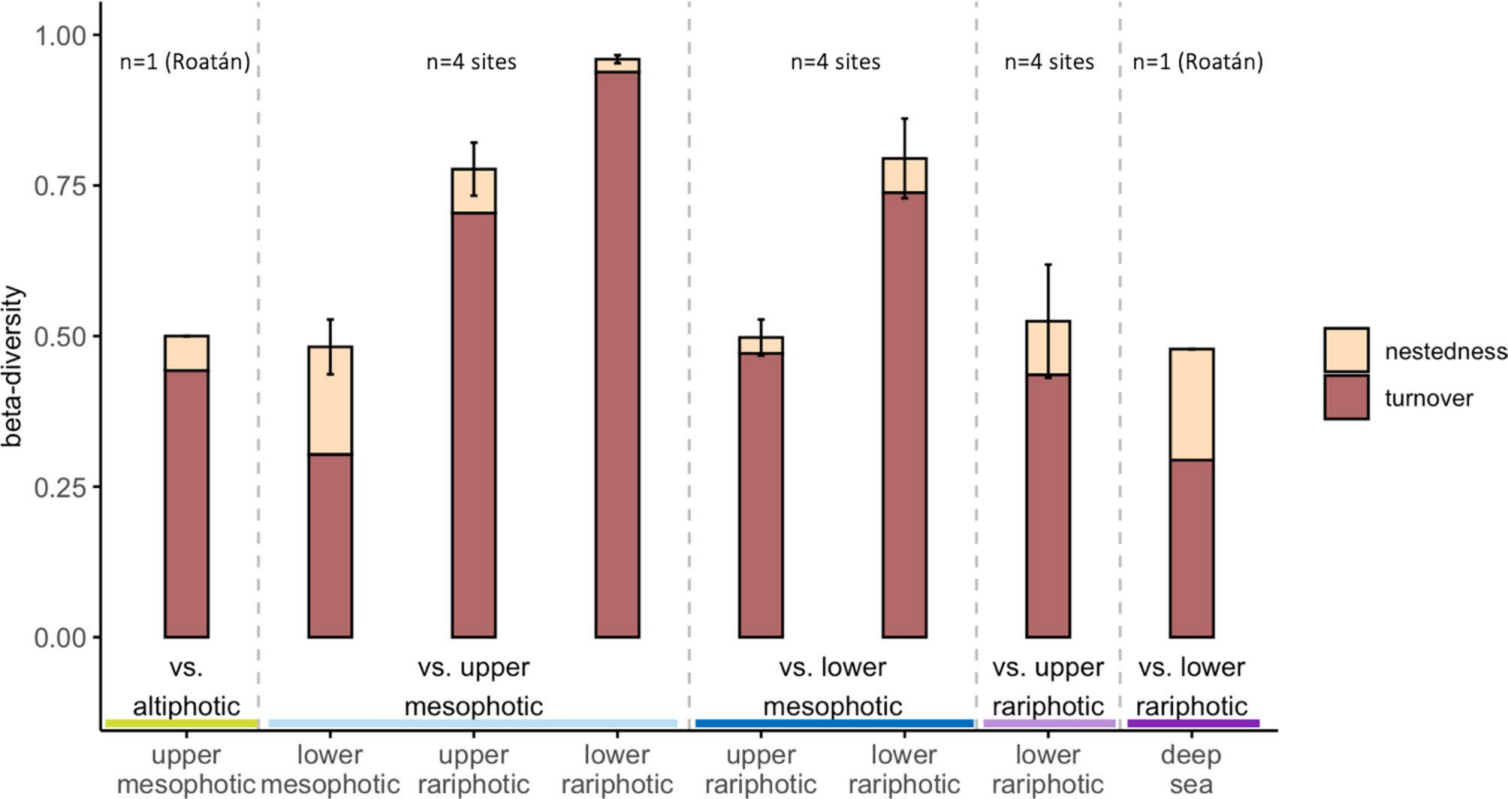
Beta-diversity and its components, nestedness and turnover, of reef-fish communities across depth zones. Values are shown as the average of values across the four sites, and error bars indicate standard error. “Altiphotic versus upper mesophotic” and “lower rariphotic versus below rariphotic” values were only available for Roatán. Coloring of bars indicate the relative contribution of nestedness (beige) and turnover (red).

Dissimilarity (i.e., total beta-diversity) between depth zones increased with depth disparity, with the highest dissimilarity (~ 100%) occurring between the upper mesophotic and the lower rariphotic (Figs. 5, S3). Dissimilarity between adjacent depth zones was ~ 50% across depths and sites and mostly resulted from species turnover. However, higher contribution of nestedness (40%) between the upper and lower mesophotic communities, and between the lower rariphotic and the next deeper depth zone in Roatán, suggest that species drop-out plays an important role in these faunal transitions.

### Depth affinities of species

**Figure 6:**
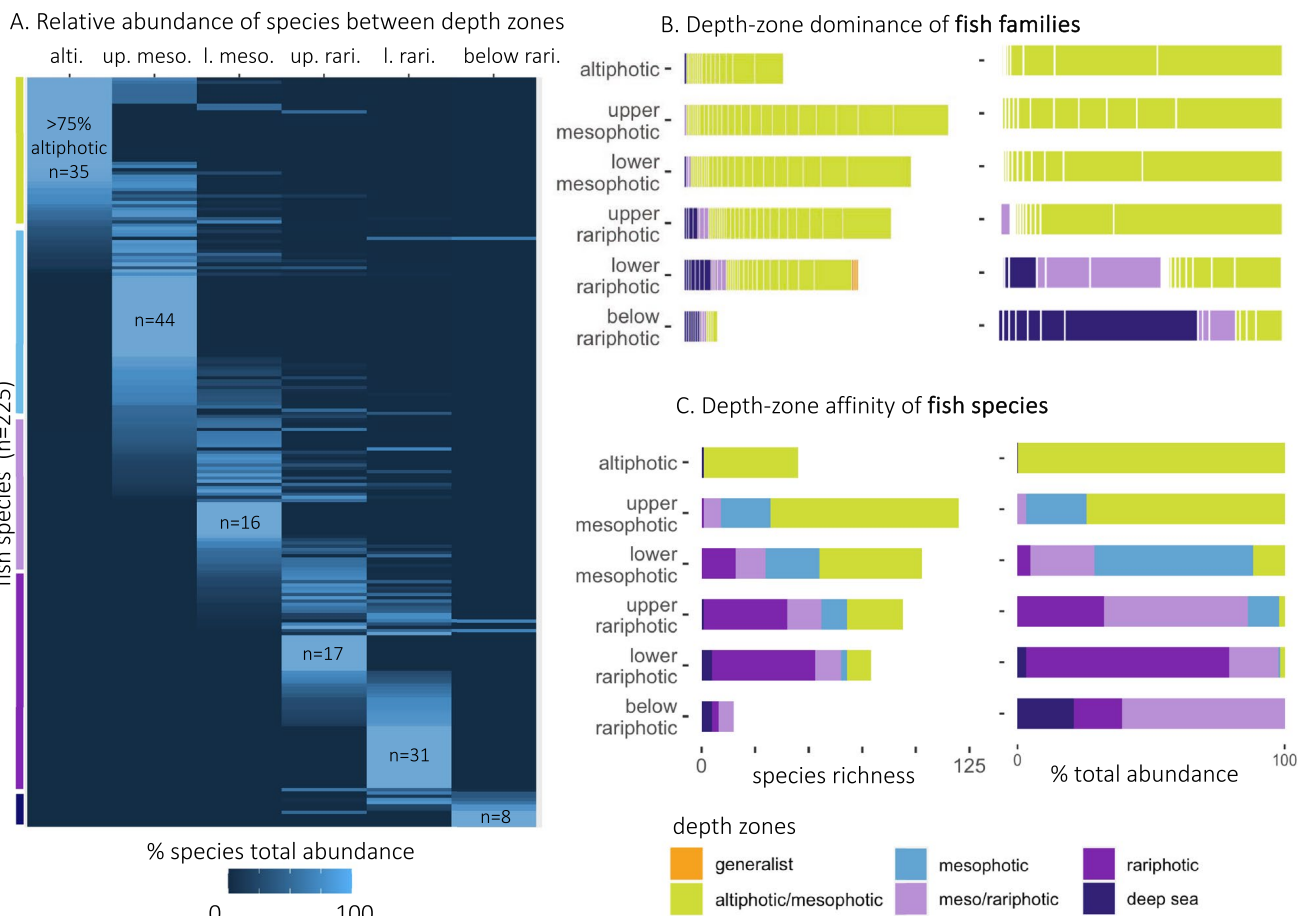
Depth affinity of reef-fish species and families across depth zones. Distribution (% total abundance) of fish species among depth zones (**A**). Each line displays the relative abundance of a given species across five depth zones from the “altiphotic” to “below rariphotic” (300–480 m). Vertical colored lines indicate species falling under each depth affinity category given their depth distribution. The number of depth specialists of each depth zone (> 75% of total abundance in a given depth zone) is indicated on the figure (“n = ”). Depth-zone dominance of fish families (**B**) and depth-zone affinity of species (**C**) in each depth zone, and contribution of depth affinity groups to species richness (left panel) and abundance (right panel). White lines in barplots of panel C distinguish species from different families. Filling colors indicate the depth affinity of species, from the altiphotic to the deep sea. Results are shown pooled across the four study locations, except for altiphotic and below rariphotic zones for which data was only collected in Roatán.

We found that 151 out of 225 species were depth specialists with > 75% of their observed abundance occurring in a specific depth zone: n = 35 in the altiphotic, n = 44 in the upper mesophotic, n = 16 in the lower mesophotic, n = 17 in the upper rariphotic, n = 31 in the lower rariphotic and n = 8 below the rariphotic (Fig. 6A). When considering depth-zone predominance at the family level, we found that reef-fish families dominated all depth zones in abundance and richness from the altiphotic to the lower rariphotic. This result stood true when pooling observations across sites (Fig. 6B) and when considering sites individually (Fig. S6). Species from altiphotic/mesophotic families dominated in abundance down to the upper rariphotic and in richness down to the lower rariphotic, but rariphotic taxa dominated in abundance in the lower rariphotic. Below the rariphotic (300–480 m), fish communities were dominated in abundance and richness by species from deep-sea families (e.g., Epigonidae, Triacanthodidae, Trachichthyidae; Table S6). However, when considering depth affinity at the species level (Fig. 6C), deep-sea fishes were only present in the lower rariphotic and below, and represented a minority of total abundance and richness even in the 300–480 m depth zone. The altiphotic, upper mesophotic, and lower rariphotic were dominated by species from a single depth-zone affinity group (altiphotic/mesophotic and rariphotic, respectively), while the lower mesophotic and upper rariphotic were characterized by overlaps between four depth-zone affinity groups (Fig. 6C). Peaks in the richness of depth-zone affinity groups occurred at different depths among sites and tended to match site-specific depth breaks identified from hierarchical clustering (Fig. S5).

### Habitat segregation by depth

**Figure 7:**
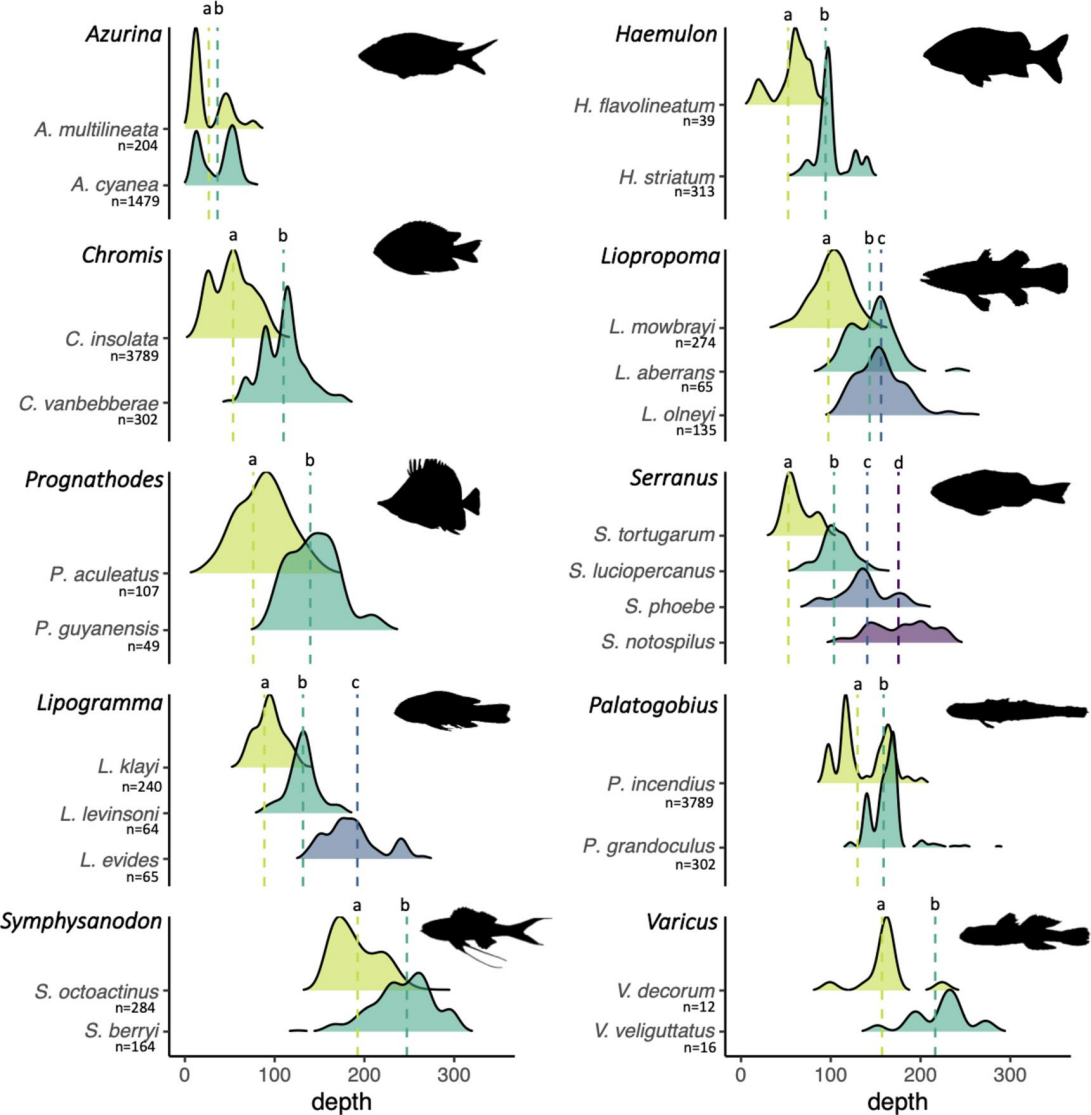
Depth segregation among congeners. Lines indicate the mean depth of occurrence of each species. Colors of abundance areas indicate species. Statistically different mean depths within a genus are indicated by different letters (Table S4). A comparison of depth distributions observed at our sampling sites versus global depth ranges is provided in Fig. S7.

We found strong evidence for depth segregation between species of the same genus (Fig. 7). Within all ten genera tested, species was a significant factor influencing mean depth of occurrence (Wilcoxon and Kruskal–Wallis tests, *p*-values < 0.01), and all pairwise comparisons of mean depths between species of a same genera were statistically significant (*p*-values < 0.04, Table S4and S5). Depth segregation occurred both between and within depth zones. For instance, three *Lipogramma* species partitioned across the upper mesophotic, lower mesophotic and upper rariphotic, while *Azurina multilineata* and *A. cyanea* displayed depth segregation within the upper mesophotic (Fig. 7). The depth distributions of species varied across sites, reflecting in some instances the variations in depth zones’ boundaries between sites (e.g., see *Palatogobius incendius*, Fig. S2).

### Cross-site community analyses

**Figure 8:**
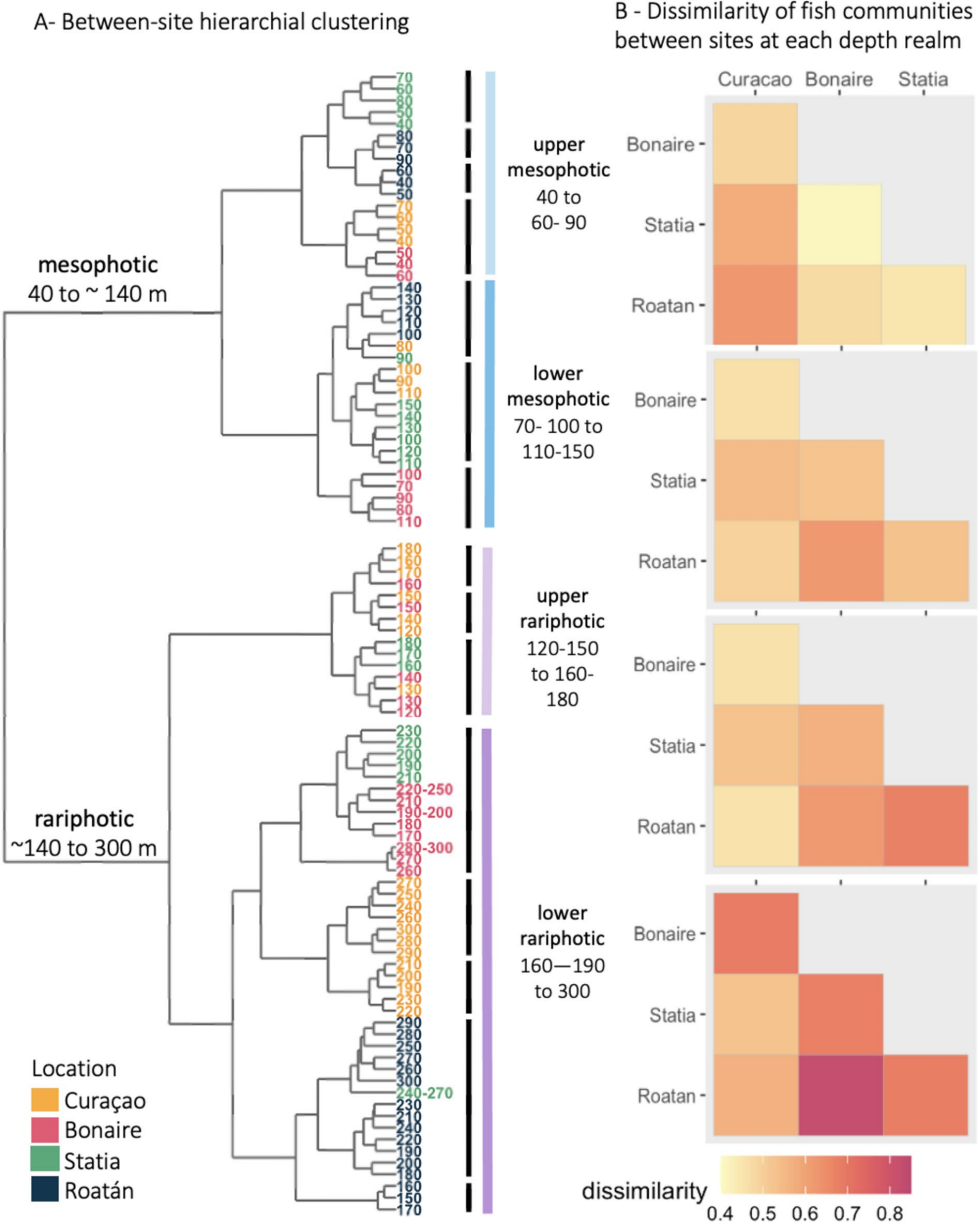
Cross-site dissimilarity analysis of deep-reef fish communities. (**A**) Cross-site hierarchical clustering analysis of deep-reef fish communities from 40 to 300 m. Length of branches in the dendrogram is commensurate to the dissimilarity between depth bins based on Bray–Curtis distance. Font color indicates sites. Significant clusters (SIMPROF analyses, Ward linkage) are indicated by thick black vertical lines. (**B**) Dissimilarity of deep reef-fish communities between sites at the four depth zones defined in this study, based on the presence/absence of fish species.

We performed a cross-site hierarchical clustering of deep-reef fish communities between 40 and 300 m using a Ward linkage algorithm (Fig. 8A). We found that the 96 fish communities corresponding to distinct sites and depth bins formed 15 significant clusters (SIMPROF based on Bray–Curtis distance, alpha = 10^–6^). The total number of significant clusters was unchanged when adding communities observed at 10–40 m and 300–480 m in Roatán (Fig. S4). The two first levels of branching of the dendrogram (Fig. 8A) reproduced the four depth zones identified at the site-level (Fig. 3). The first level of branching occured 120 to 160 m deep depending on site, marking the transition between the mesophotic and the rariphotic. The second level of branching within the mesophotic branch (40 to 120–160 m) formed two groups with a depth break at 70–100 m depending on site, marking the transition between the lower and upper mesophotic. The second level of branching within the rariphotic branch (120–160 to 300 m) formed three groups: one group encompassing all rariphotic depths from Roatán (150–309 m), and two groups separating the lower and upper rariphotic depth zones from Curaçao, Bonaire and Statia, with a depth break at 170–190 m. The depth of community breaks found in the cross-site clustering (Fig. 8A) were similar to those found in site-specific clustering (Fig. 3).

We found that depth zone, site location, and the interaction between these two factors had a significant influence, in this order, on fish community structure (PERMANOVA, Table S7). In the upper mesophotic, hierarchical clustering indicated greater similarities between Curaçao and Bonaire on one hand, and Roatán and Statia on the other (Fig. 8A). However, in the lower mesophotic, fish communities from Bonaire and Roatán pooled separately, while Statia and Curaçao pooled together. In the upper rariphotic, communities from Curaçao, Bonaire, and Statia were pooled together while fish communities from Roatán were in a separate cluster. Beta-diversity based on the presence/absence of species confirmed that similarity between sites varied across depth, with Roatán tending to display higher dissimilarity to other sites (Fig. 8B). Cross-site beta-diversity values remained within similar ranges from the upper mesophotic to the upper rariphotic but were higher in the lower rariphotic.

## Discussion

### Vertical zonation of reef-fish communities

The present study provides an extensive analysis of the vertical structure of reef fishes based on continuous, direct visual observations of communities from 40 to 300 m depth at four Caribbean sites, with additional data from 10 to 480 m depth at one of these site. The number of species observed per site (73–145) is within the range found by previous deep-reef fish studies in the Caribbean (e.g., 103 in Puerto Rico^24^). The patterns of decreased richness and abundance with depth documented in this study are consistent with trends observed in the Caribbean^25^ and other regions of the world^6,7^. While we found that the number of distinct community clusters and their depth boundaries varied between sites (Fig. 3), we identified four depth zones occurring at all sites: the upper mesophotic (~ 40 to 90 m), the lower mesophotic (~ 100 to 140 m), the upper rariphotic (~ 150 to 190 m), and the lower rariphotic (~ 200 to 300 m). This depth zonation was found both using site-specific hierarchical clustering (Fig. 3) and cross-site hierarchical clustering (Fig. 8). The only exceptions were the deep-reef fish communities from Statia for which the distinction between lower mesophotic and upper rariphotic was more ambiguous. Greater similarity between the upper and lower mesophotic on one hand, and between the upper and lower rariphotic on the other hand, was supported by the branching hierarchy of the cross-site dendrogram (Fig. 8A). Our results support a two-tier vertical zonation nomenclature for deep reefs as established in Baldwin et al.^10^ with a first level comprising two zones (the mesophotic and rariphotic) separated by a strong community break, and a secondary level separating “upper” and “lower” communities within these zones. While the 300 m lower boundary of the lower rariphotic was originally set by the depth limitations of submersibles rather than by ecological observations^7,10^, the depth break found at 300 m in Roatán between the lower rariphotic and a deeper fish community that extends down to at least 480 m now supports the ecological reality of this boundary. To the best of our knowledge, the only other study that tested the lower limit of the rariphotic identified a comparable faunal break depth at 320 m in Hawaii^8^.

The depth of community breaks we identified match those of previous studies conducted locally, especially by Pinheiro et al.^17^ who documented a strong fish species turnover at 80 m in Curaçao, matching the upper to lower mesophotic transition found here. The few studies conducted outside of the Caribbean over a comparable depth range have also reported successions of three to five distinct reef-fish assemblages^8,26^, but differences in the number of distinct assemblages and in the depth of faunal breaks suggest important interregional variability. The many more studies that have investigated the vertical zonation of deep-reef fish communities have been limited to a maximal depth of 60–100 m; as such, they have only captured the upper range of deep reefs^9,12^. Conversely, some studies have extended below 300 m depth but did not describe fish communities continuously across depths, hindering depth-zonation analyses^11,27^. As a result, our understanding of the number of depth zones and their boundaries across oceanic regions remains limited. Given that the shallowest deep-reef fish community break generally occurs around 60–100 m ^10,26,28^, surveys extending down to at least 150 m are necessary to adequately locate deep community breaks. In that regard, submersible surveys combined with the use of ichthyocides or anesthetics offers unparalleled means to describe deep-reef fish communities. In particular, only submersibles allow for direct visual observation and fish collection below ~ 150 m, the lower limit of rebreather diving. Such sampling techniques are necessary to document cryptic species, a major component of deep-reef fish communities that cannot be readily observed using remotely operated vehicles or underwater video systems^4,29^.

### Shifts in fish community structure across depths

We found high and relatively constant values of dissimilarity (~ 50%) between fish communities of adjacent depth zones resulting mostly from species turnover (Fig. 5), corroborating existing evidence for the ecological uniqueness of deep-reef fish communities^2,22,26^. While previous studies have reported important taxonomic turnover between deep-reef fish communities^22,26^ only one study has tested this down to rariphotic depths^26^. While Semmler et al.^26^ reported lower rates of turnover at rariphotic than at mesophotic depths, we found the opposite trend, with the upper to lower mesophotic transition having the lowest rate of species turnover.

We found that “altiphotic/mesophotic” species contributed to ~ 60% of total diversity and ~ 80% of total abundance in the upper mesophotic (Fig. 6C). This aligns with previous studies demonstrating that a large proportion (~ 64%) of shallow reef fishes occur down to 60 m ^9^ and suggests that upper mesophotic fish communities are largely composed of shallow reef fishes at the extremity of their depth distribution. The ecological continuum between altiphotic and upper mesophotic fish communities was further supported by SIMPROF analyses which identified a single cluster from 20 to 60 m in Roatán (Fig. 2). These results suggest that the upper mesophotic boundary typically considered at around 30–40 m in the literature might reflect conventional SCUBA limits, as well as breaks in habitat-forming communities, rather than actual fish community breaks. By contrast, the high rates of rariphotic specialists in the rariphotic depth zones (Fig. 6C) combined with the important species turnover found between mesophotic and rariphotic communities (Fig. 5) confirm the ecological uniqueness of rariphotic fish communities, a depth zone only recently described by Baldwin et al.^10^. Together, these results suggest that the faunal break between the upper and lower mesophotic is numerical (i.e., decrease in abundance and loss of species) rather than compositional (i.e., species turnover), and represents a weaker faunal break than the mesophotic to rariphotic transition. We also confirm that the rariphotic zone is dominated by reef-affiliated families in the Caribbean (Fig. 6B), a result previously documented only in Curaçao^10^. Lastly, we determine that the lower boundary of the rariphotic occurs at 300 m in Roatán and corresponds to the depth limit of communities dominated by reef-affiliated fish families. From 300 to 480 m, we found a single community dominated by rariphotic specialists from deep-sea families rather than true deep-sea species.

### Depth specialization and segregation in reef fish taxa

We found that the majority of fish species observed at our study sites occur predominantly within a single depth zone (Fig. 6A). Eighty-five percent of deep-reef species (i.e., excluding altiphotic and deep-sea affiliated species) were depth specialists, meaning that > 75% of their total abundance was confined to either mesophotic or rariphotic depths. Furthermore, our study documents depth segregation among ten reef-fish genera, with up to four species of a given genus occurring at distinct average depths (Fig. 7). These results demonstrate that habitat segregation by depth is an important ecological mechanism driving the vertical zonation patterns observed on reefs. Moreover, depth segregation may be important in driving speciation in reef fish, or at the very least, an important factor in maintaining species boundaries. A substantial part of deep-reef fish diversity originates from speciation of shallow-reef fish having adapted to deep-reef environments^10,30^. Depth segregation has already been demonstrated on deep reefs for the family Gobiidae^30,31^, the genus *Lipogramma*, and the genus *Lipropoma*^32,33^, or the genus *Bathypterois* found on seamounts^11^. Importantly, large differences between global depth distributions and locally realized depth distribution (Fig. S9) suggests that depth segregation processes are underestimated when working with global datasets.

### Regional variability of deep-reef fish communities

We found that fish communities from a given depth zone shared many characteristics across sites. The most abundant species at a given depth zone were usually found at several sites and participated in driving community distinctiveness between depth zones (Fig. 4). Some of these key species have already been identified at different Caribbean sites^7,11^, in particular *Pronotogrammus martinicensis* and *Serranus phoebe* as indicators of rariphotic communities, and *Paranthias furcifer* as an indicator of mesophotic communities. Such indicator species could be useful to more promptly identify vertical boundaries of depth zones at new study sites, or to test whether depth boundaries at a given site are changing over time. The limited coastal extent (~ 1 km) sampled at each island precludes us from explaining differences in fish communities between sites through a biogeographic lens. Rather, variations in vertical zonation and in fish-community structure are likely due to local characteristics of study sites, such as irradiance, temperature, nutrient availability^34^, and habitat characteristics^6,7,13^. Local geomorphological specificities of study sites, such as the location of reef walls and reef flats, could explain a large part of the variability in vertical zonation and community structure of deep-reef fish^15,35^. Human impacts, particularly fishing pressure and coastal development, could also explain differences observed between sites^36^. Notably, the survey site at Bonaire occurs within a marine protected area^37^ while the study site in Curaçao is located in front of a resort complex and experiences fishing and shoreline artificialization. More research is needed to document how such pressures affect deep-reef fish communities^21,38^.

### Increased endemism in the rariphotic?

The higher regional beta-diversity in the lower rariphotic revealed in this study could be due to (1) lower fish abundance, which might have amplified biases associated with incomplete sampling effort by artificially increasing differences between rariphotic communities described, (2) greater influence at rariphotic depths of local environmental variables and micro-habitats on community structure, (3) higher rates of endemism at rariphotic depths, or (4) a combination of these factors. While assessments of endemism rates at rariphotic depths are unprecedented, higher rates of endemism on mesophotic reefs than on shallow reefs have been documented by an increasing body of literature^18,39,40^, and surveys of deep reefs across the Pacific found that deep-reef fishes tend to have smaller geographic ranges than shallow-reef fishes^39,41^. The greater environmental stability of deep reefs, in particular in relation to sea-level change associated with glacial-interglacial cycles^42^, has been hypothesized to promote higher rates of endemism at depth^39^, and could explain how endemism occurs on Caribbean deep reefs despite extremely low regional levels of endemism on Caribbean shallow reefs^43^. While close to half (48 out of 109) of mesophotic and rariphotic species observed in this study were found at a single site, it is currently unclear whether these species are locally endemic. Extending deep-reef studies to new sites across ocean basins will help us to determine whether increased rates of endemism are indeed a characteristic of deep-reef fish communities or rather reflect decreasing sampling efforts with depth.

### Accounting for depth in biogeographic studies

Decades of biogeographic research have attempted to divide the world’s tropical reef regions into homogeneous and distinct ecological units^43,44^. The resulting classifications are based almost exclusively on the distribution of shallow-reef species and, as such, only account for ~ 20% of reef biodiversity. Given the growing evidence that deep-reef communities exhibit distinct diversity patterns relative to shallow reefs^16^ and could be influenced by different diversification mechanisms^39^, we suggest that the delimitation of marine realms and ecoregions should shift from a two-dimensional (2D) to a 3D approach. Adding depth boundaries to marine ecological units would reflect that distinct marine communities co-occur at a given location at different depths. For example, “depth zones” corresponding to distinct ecological communities, and “depth realms” corresponding to distinct taxonomic communities (e.g., reef-affiliated vs. deep-sea affiliated) could constitute two levels of subdivision of marine units across depth. At minima, we suggest that biogeographic studies revisit current classification by incorporating the rapidly evolving data on the distributional range of deep-reef and deep-sea species^45^. Similarly, biogeographic theories developed to explain spatial patterns of reef-fish diversity, such as the center of overlap, origin, and accumulation hypotheses^46,47^, have been restricted to explaining diversity patterns of shallow-reef fishes. Testing whether these theories could be transposed to explain diversity gradients across both space and depth could represent the next step for reef-fish biogeographic studies.

## Conclusion

Continuous observations from the surface to 480 m at four sites in the Caribbean suggest that six vertically-stratified fish communities occur across these depths, including five reef-fish dominated communities down to 300 m. Vertical community zonation was comparable across sites suggesting regional stability of this pattern, although we observed variations in the dominating species and in the exact depth of community breaks, likely a result of local environmental conditions. Our results demonstrate that several ecologically distinct deep-reef fish communities occur below shallow reefs and support the growing literature calling to extend conservation actions beyond shallow ecosystems to ensure that the full succession of marine diversity benefits from protection.

## Methods

### Sites

Data collection occurred at four locations in the Caribbean (Fig. 1): two neighboring western Caribbean islands that are part of the Lesser Antilles (Bonaire and Curaçao), an eastern Caribbean island that is part of the Leeward Islands (Saint Eustatius, hereafter Statia), and a western Caribbean island off the coast of mainland Honduras (Roatán). See Supplementary Information for a more detailed description of islands’ environmental and ecological specificities.

### Data collection

*Submersible transects.* All fish data were collected using human-occupied submersibles as part of the Smithsonian Deep Reef Observation Project (DROP). Fishes were identified by two trained observers (among Luke Tornabene, Ross Robertson, and Carole Baldwin) in the submersible as it slowly descended (first half of dives) or ascended (second half of dives) the reef slope. Ascents were conducted on a different trajectory than descending transects. Some fishes, with emphasis on cryptobenthic fishes, were opportunistically collected using anesthetics and a suction hose attached to the submersible, either for identification purposes or for systematic projects. See Baldwin et al., (2018) for additional details.

In **Curaçao** (site coordinates: 12.083197N, 68.899058W) fish data were collected between 2011 and 2016 for a total of > 100 submersible dives ranging from 40 to 310 m using the Curasub. Surveys covered ~ 1000 m on both sides of the drop-off site. In **Bonaire,** fish data were collected in January 2017 during 11 submersible dives. Submersible dives were performed between Klein Bonaire Island and the South of Punt Vierkant by Belnem (drop-off coordinates: 12.094974N, 68.296635W) between 40 to 300 m depth using the Curasub. Each dive averaged 3–4 h in duration and altogether covered ~ 1,000 m on both sides of the drop-off site. Depths between 40 and 150 m were systematically covered, five dives extended down to 180–230 m, and one dive reached 300 m. In **Statia,** fish data were collected in April 2017 during 12 submersible dives between 40 and 305 m depth using the Curasub. Different locations off the southwestern edge of the island were sampled using the R/V Chapman (Robertson et al., 2020), including Gallows Bay (17.474734N, 62.987370W) and Kay Bay (17.467653N, 62.978778W). Dives were conducted off the southwestern edge of the island’s 200 m platform and lasted 5–6 h. In **Roatán** (drop-off coordinates: 16.305557N, 86.597669W), fish data were collected between 2016 and 2018 during 17 dives from 0 to 480 m using the submersible Idabel. Each dive averaged 3–4 h in duration, and altogether covered ~ 500 m on both sides of the drop-off site. *Guidelines for the Use of Fishes in Research* co-established by the American Society of Ichthyologists and Herpetologists^48^ were followed for all field-collecting activities, and fish specimens collected as part of this study were done so under Smithsonian Animal Care and Use Committee (ACUC) approval to C. C. Baldwin (ACUC #2011–07 and #2014–13). No In Vivo Experiments were conducted as part of this research.

### Data analysis

All data analyses were performed using R version 4.4.1^49^ in R Studio.

*Abundance and species richness.* Fish abundance was reported as the total number of individuals observed at each 10 m depth bin from 40 to 300 m (e.g., all observations from 40 to 49 m were grouped into the 40 m depth bin) across all submersible dives at a given site. Because sampling effort *t* (hours spent diving) per depth bin *x* varied depending on technical and environmental factors (e.g., steepness of the slope), abundance values were normalized using coefficients denoting the relative time spent at each depth bin *x* and site *z* (Baldwin et al., 2018):

1 $$\text{ abundance normalized}_{x,z} = \text{abundance}_{x,z} \times \text{sampling coefficient}_{x,z}$$

with sampling coefficient at depth *x* of site *z* being calculated as:

2 $$\text{sampling coefficient}_{x,z} = \frac{{ \mathop {\max }\limits_{x} (t_{x,z} )}}{{t_{x,z} }}$$

Because total sampling time also varied between sites, we transformed normalized abundance values into relative abundance values when comparing data between sites (see Fig. 2):

3 $$\text{relative abundance} \left( x \right) = \frac{{ \text{abundance normalized}_{x} }}{{\mathop {\max }\limits_{{x \in \left[ {40,300} \right]}} \left( {\text{abundance normalized}_{x} } \right)}} \times 100$$

Models of abundance and species richness were fitted using generalized additive models ({mgcv} package, gam() function) accounting for depth and location (abundance ~ depth + location. We used Poisson error distributions to account for the non-normal data structure, accounting for depth and location (abundance ~ depth + location).

*Community structure.* The depth structure of fish assemblages was examined using multivariate hierarchical clustering methods. First, we calculated the Bray–Curtis dissimilarity matrix, which accounts for species presence and their relative abundance within the community. To avoid overemphasizing the importance of rare species in our analysis, while also controlling for extremely abundant species that sometimes occur in large schools, we applied a square-root transformation to abundance data before performing cluster analyses. Additionally, depth bins for which fewer than five individuals were observed were pooled with the next deeper 10 m depth bin until a collective total of five individuals was reached. This was done to counter biases arising from the fact that there are inherently fewer similarities between communities with fewer individuals, as in^50^. We used a complete-linkage clustering algorithm, which seeks to maximize distance between clusters based on the two most distant objects in each cluster, to group depth bins based on species composition. Hierarchical cluster dendrograms and nonmetric multidimensional scaling ordinations (MDS) based on Bray–Curtis distances were used to visualize community structure. The number of significantly distinct depth clusters was determined using Analysis of Similarity Profiles (SIMPROF)^51^ based on a complete linkage algorithm, Bray–Curtis distances, and a conservative value of alpha = 10^−7^ as in^11,52^. Based on main branching events in dendrograms, we pooled adjacent clusters into six depth zones: altiphotic, upper mesophotic, lower mesophotic, upper rariphotic, lower rariphotic, and “below rariphotic” (see Fig. S8). We confirmed the distinctiveness of these depth zones a posteriori using PERMANOVA^53^. We used a similarity percentage analysis (SIMPER)^53^ to determine which species contributed most to differences between adjacent depth zones. Lastly, we tested for faunal depth breaks across sites by performing a hierarchical clustering and SIMPROF analyses on depth bins across all four sites based on a Ward linkage (which forms clusters by minimizing variance within clusters), Bray–Curtis distances, and alpha-value = 10^−7^.

*Depth segregation.* To test whether depth is a driver of niche segregation between closely related species, we compared the depth distribution of species belonging to the same genus. This was performed for all genera for which at least two species had been observed on deep reefs, with each species represented by a minimum of ten individuals across study sites. Because the depth distribution of species from most genera tested (eight out of ten) did not follow a normal distribution (Shapiro–Wilk normality test, *p* < 0.05) we performed Kruskall–Wallis tests followed by pair-wise Wilcoxon tests to assess the correlation between species and depth (depth ~ species). Statistical tests were performed on original depth observations (non-transformed for sampling effort), but density curves and means represented in Fig. 7 display abundance corrected for sampling effort (as in Eq. 1).

*Depth affinities of fishes*. We defined five categories of depth affinities at the species level: “altiphotic/mesophotic”, “mesophotic”, “mesophotic/rariphotic”, “rariphotic”, and “deep-sea”. A species was determined to have an affinity for a given depth zone when ≥ 75% of observations^10^ occurred in that zone. We classified species as belonging to the “altiphotic/mesophotic” category when ≥ 75% of observations in this study occurred at mesophotic depths, but the species was also known to commonly occur above 40 m depth^54,55^. Similarly, the “deep-sea” depth affinity was attributed to species typically associated with deep-sea ecosystems, but also observed in the present study (e.g., *Synagrops spp.* , *Bathyclupea sp*. , *Epigonidae sp.*; see Table S1 for complete list). When determining in which depth zones a species was observed, we used the site-specific depth breaks from hierarchical clustering analyses (Fig. 2).

We further defined four categories of depth dominance at the family level based on published fish databases^54–56^ and expert knowledge: “altiphotic/mesophotic”, “rariphotic”, “deep-sea”, and “depth generalists” (Table S1). We classified families as “altiphotic/mesophotic” when the depth ranges of their members were predominantly shallower than 130 m, as rariphotic when the depth ranges of their members were predominantly between 130 and 500 m, and as deep-sea affiliated when the depth ranges of their members were predominantly below 500 m. In addition to these three categories first defined by Baldwin et al., (2018), we classified families as depth generalists when their members spanned multiple depth zones, which was the case for some non-reef affiliated families (e.g., Paralichthyidae).

*Dissimilarity across depths and between sites.* We calculated the β-diversity index ${\beta }_{sor}$^57^ between depth zones to assess whether the distinctiveness of deep-reef fish communities is predominantly driven by changes in species composition (i.e., turnover) or by loss in diversity (i.e., nestedness^58^):

$$\beta_{sor} = \frac{b + c}{{2a + b + c}} \,\,\,\,\,\,\,\,\,\,\,\,\,\,\,\,\,\,\,\,\,\,\,\,\,\,\,\,\,\,\,\,\,\,\,\,\,\,\,\,\,\,\,\,\,\,\,\,\,\,\,\,\,\,\,\,\, \,\,\,\,\,\,\,\,\,\,\,\,\,\,\,\,\,\,\,\,\,\,\,\,\,\,\,\,\,\,\,\,\,\,\,\,\,\,\,\,\, \,\,\,\,\,\,\,\,\,\,\,\,\,\,\,\,\,\,\,\,\,\,\,\,\,\,\,\,\,\,\,\,\,\,\,\,\,\,\,\,\, \,\,\,\,\,\,\,\,\,\,\,\,\,\,\,\,\,\,\,\,\,\,\,\,\,\,\,\,\,\,\,\,\,\,\,\,\,\,\,\,\, \left( 4 \right)$$,

where *a* is the number of species common to both groups (site or depth bin), *b* is the number of species that occur in the first group but not in the second, and *c* is the number of species that occur in the second group but not in the first. This index varies between 0 and 1, with 1 indicating no species in common between sites and 0 indicating identical species composition between sites.

We calculated the turnover component of dissimilarity using the Simpson dissimilarity index (β_sim_), which calculates the amount of dissimilarity due to the presence of distinct species in both groups:

5 $$\beta_{sim} = \frac{{min\left( {b,c} \right)}}{{a + min\left( {b,c} \right)}}$$

β_sim_ varies between 0 and 1, with 1 denoting that no species are shared between the two groups, and 0 denoting that all species from a group correspond to a sub-sample of the species from the other group.

The nestedness component of pairwise dissimilarity, i.e., the component of dissimilarity linked to species loss rather than species turnover, is calculated by subtracting $\beta sim$ from $\beta sor:$

6 $$\beta_{nes} = \beta_{sor} - \beta_{sim}$$

Taxonomic dissimilarity, nestedness, and turnover components were computed in R using the {betapart} package^59^.

### Ethics approval

Fish collections in Curaçao and Bonaire were conducted under research permits granted to Substation Curaçao. Permits for fieldwork in Roatán were approved under research permit number DG/PMM 010-2018. All collections were made in accordance with University of Washington IACUC protocol #PROTO201800092.

### Supplementary Information

Supplementary Information.

## Data Availability

All data and R scripts used for analyses are available through the author’s personal GitHub repository (https://github.com/jjacquemont/Deep.Reef.Fish.Carib).
