# Supplementary material for: Vertical structure of Caribbean deep-reef fishes from the altiphotic to deep-sea boundary
Source: Sci Rep. 2024 Aug 22;14:19489. doi: 10.1038/s41598-024-69774-w (PMC11341716; doi:10.1038/s41598-024-69774-w)
Supplement: Supplementary file 1 — Supplementary Information. [file 41598_2024_69774_MOESM1_ESM.pdf]

Vertical structure of Caribbean deep-reef fishes from the altiphotic to deep-sea boundary

Juliette Jacquemont, Simon J. Brandl, Emily P. McFarland, Joachim Claudet, Carole C. Baldwin, Jenna Barrett, Luke Tornabene

Supplementary information

Study sites.

Curaçao and Bonaire are two neighboring oceanic islands in the westernmost part of the Lesser Antilles, situated 60 km north of Venezuela. Islands are separated from the South American mainland and from each other by a deep-water trench (4000 - 5000 m) from which they experience strong influence of deep oceanic waters (Frade et al., 2019). Both islands experience a semi-arid climate, with average annual precipitation below 500 mm and sea surface temperature varying between 26 and 29 °C . Both islands are surrounded by steep fringing reefs on their leeward sides and intermittent patches of mangrove, and display some of the best coral cover of the Caribbean (Jackson et al., 2014). Bonaire and Curaçao harbor among the best studied mesophotic ecosystems of the world, thanks to ongoing research conducted since the 1970s, a result of easily accessible deep reefs and favorable geopolitical context (Frade et al., 2019). Curaçao is the largest island of the Dutch West Indies at 61 km long and 14 km wide, for a total surface of 444 km^2. The island is surrounded by a fringing reef 20–250 m from the coast, covering a total surface of 7.85 km^2 (Pinheiro et al., 2016). The estimated mean coral cover in Curacao averages 20-30 % on shallow southern reefs where the submersible dives were conducted (Jackson et al., 2014). Bonaire is located immediately East of Curaçao. It is 40 km long and 12 km wide, for a total surface of 288 km^2, and displays coral cover of 38%.

Sint Eustatius (Statia) is a 21 km^2 island situated in the northeastern Caribbean Sea. Statia is surrounded by a narrow 200 m shelf, which is most extensive on the leeward, western side. Statia displays low habitat diversity and poorly developed, low-relief coral reefs, in part due to the lack of deep embayment on the sheltered western side, required for the development of fringing and back-reefs (Robertson et al., 2020). At the sampling site a shallow reef flat extends to ~40-50 m depth before transitioning into the outer reef slope. All waters from the coast to the 30 m isobath are part of the St. Eustatius Marine Park, but only two reserves within the park are designated as no-take and no-anchoring. Shallow reef fish from Statia have been extensively sampled and are described in Robertson et al. (2020).

Roatán is an elongated island of ~ 85 km^2 offshore Honduras, which is part of the Mesoamerican Barrier Reef system. Roatan displays high habitat diversity, notably a combination of fringing- and barrier reefs separated from the island by lagoons, and extensive mangroves. The entire island is surrounded by a narrow ~1 km wide shelf. At the sampling site off Half-moon Bay, West End, a shallow lagoonal reef from 0 to ~20 m gives way to a steep (sometimes vertical) outer slope that extends down to >700 m within 2 km from shore.

These four sites fall within the same biogeographical province of the Greater Caribbean, commonly referred to as the Central Province, although Bonaire and Curaçao are at the very edge of this province and are sometimes associated with the Southern upwelling-affected Province of the Caribbean (Robertson & Cramer, 2014; Spalding et al., 2007). These sites represent three distinct ecoregions within the Great Caribbean province (Robertson and Cramer, 2014; Spalding et al., 2007): the Southern Caribbean (Curaçao and Bonaire), the Eastern Caribbean (Statia), and the Western Caribbean (Roatán).

References

Frade, P. R., Bongaerts, P., Baldwin, C. C., Trembanis, A. C., Bak, R. P. M., & Vermeij, M. J. A. (2019). Bonaire and Curaçao. In Y. Loya, K. A. Puglise, & T. C. L. Bridge (Eds.), *Mesophotic Coral Ecosystems* (Vol. 12, pp. 149–162). Springer International Publishing. https://doi.org/10.1007/978-3-319-92735-0_9

Jackson, E. J., Donovan, M., Cramer, K., & Lam, V. (2014). *Status and trends of Caribbean Coral Reefs: 1970-2012*. Global Coral Reef Monitoring Network, IUCN.

Pinheiro, H. T., Goodbody-Gringley, G., Jessup, M. E., Shepherd, B., Chequer, A. D., & Rocha, L. A. (2016). Upper and lower mesophotic coral reef fish communities evaluated by underwater visual censuses in two Caribbean locations. *Coral Reefs*, *35*(1), 139–151. https://doi.org/10.1007/s00338-015-1381-0

Robertson, D. R., & Cramer, K. L. (2014). Defining and Dividing the Greater Caribbean: Insights from the Biogeography of Shorefishes. *PLoS ONE*, *9*(7), e102918. https://doi.org/10.1371/journal.pone.0102918

Robertson, D. R., Estapé, C. J., Estapé, A. M., Peña, E., Tornabene, L., & Baldwin, C. C. (2020). The marine fishes of St Eustatius Island, northeastern Caribbean: An annotated, photographic catalog. *ZooKeys*, *1007*, 145–180. https://doi.org/10.3897/zookeys.1007.58515

Spalding, M. D., Fox, H. E., Allen, G. R., Davidson, N., Ferdaña, Z. A., Finlayson, M., Halpern, B. S., Jorge, M. A., Lombana, A., Lourie, S. A., Martin, K. D., McManus, E., Molnar, J., Recchia, C. A., & Robertson, J. (2007). Marine Ecoregions of the World: A Bioregionalization of Coastal and Shelf Areas. *BioScience*, *57*(7), 573–583. https://doi.org/10.1641/B570707

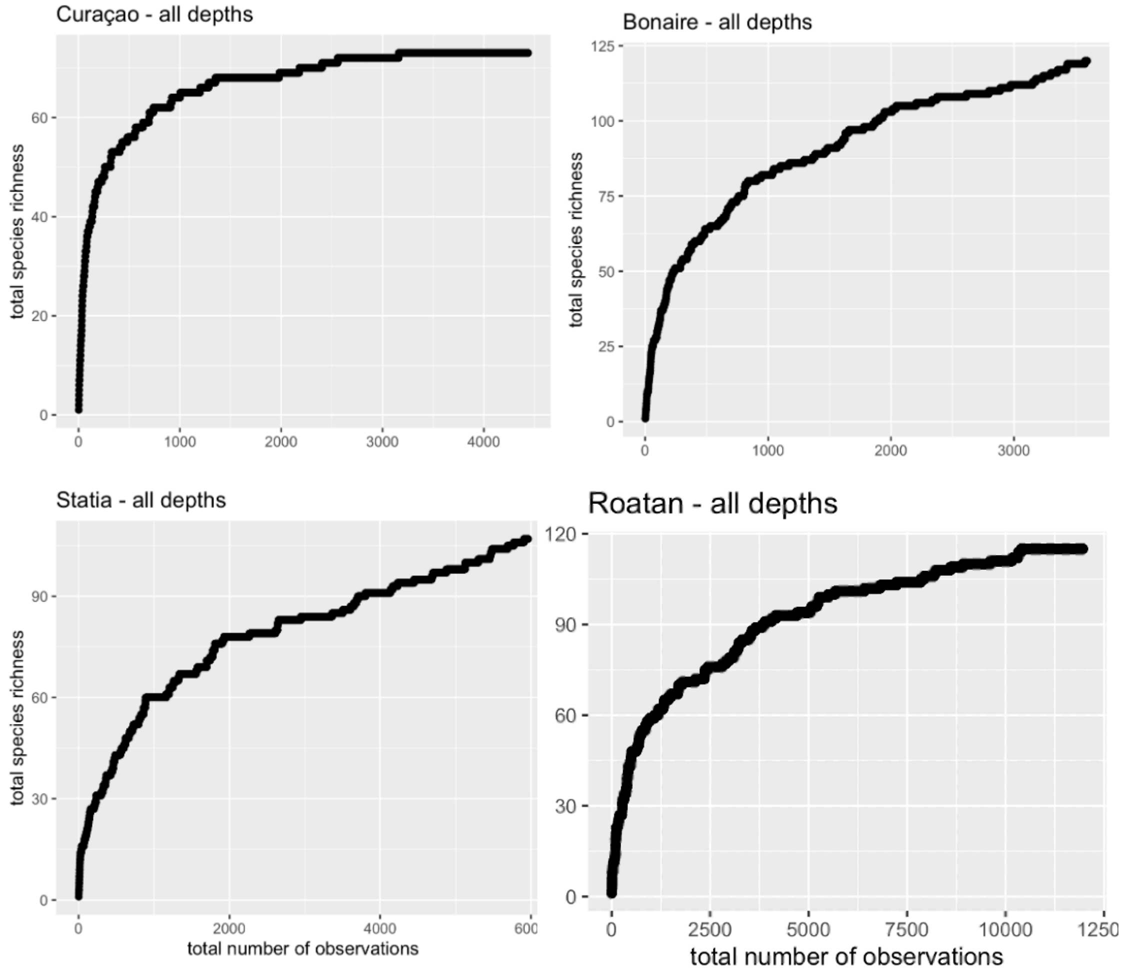

Figure S1: Rarefaction curves associated with the sampling effort of deep-reef fish diversity at the four studied sites, between 40 and 300 m.

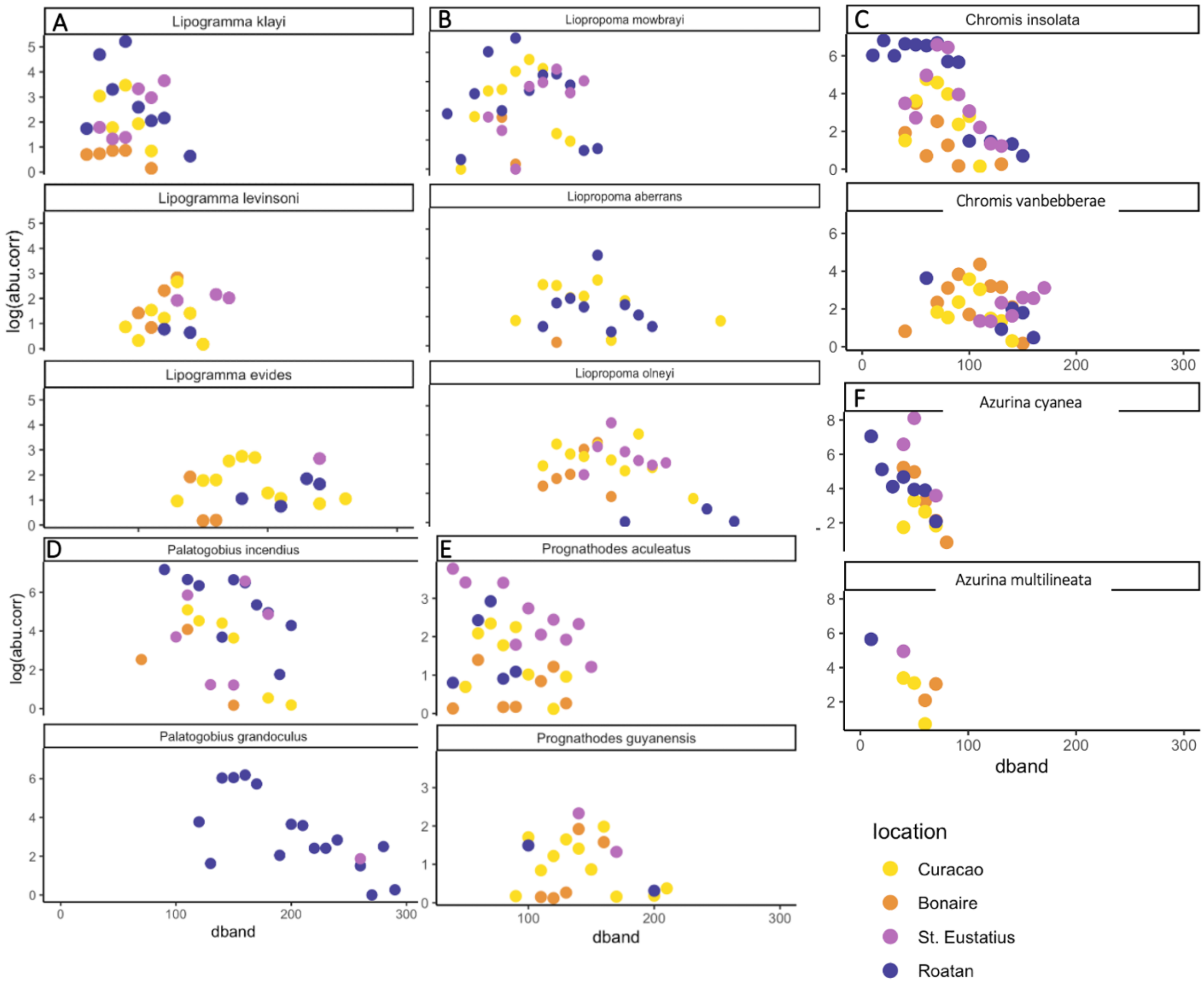

Fig S2. Depth distribution of congeners at the four study sites. Each dot represents abundance (log-transformed) at a given 10 m depth bin and location. Color of dots indicate location. The genera investigated from left to right and top to bottom are *Lipogramma* (A), *Liopropoma* (B), *Chromis* (C), *Palatogobius* (D), *Prognathodes* (E), *Azurina* (F).

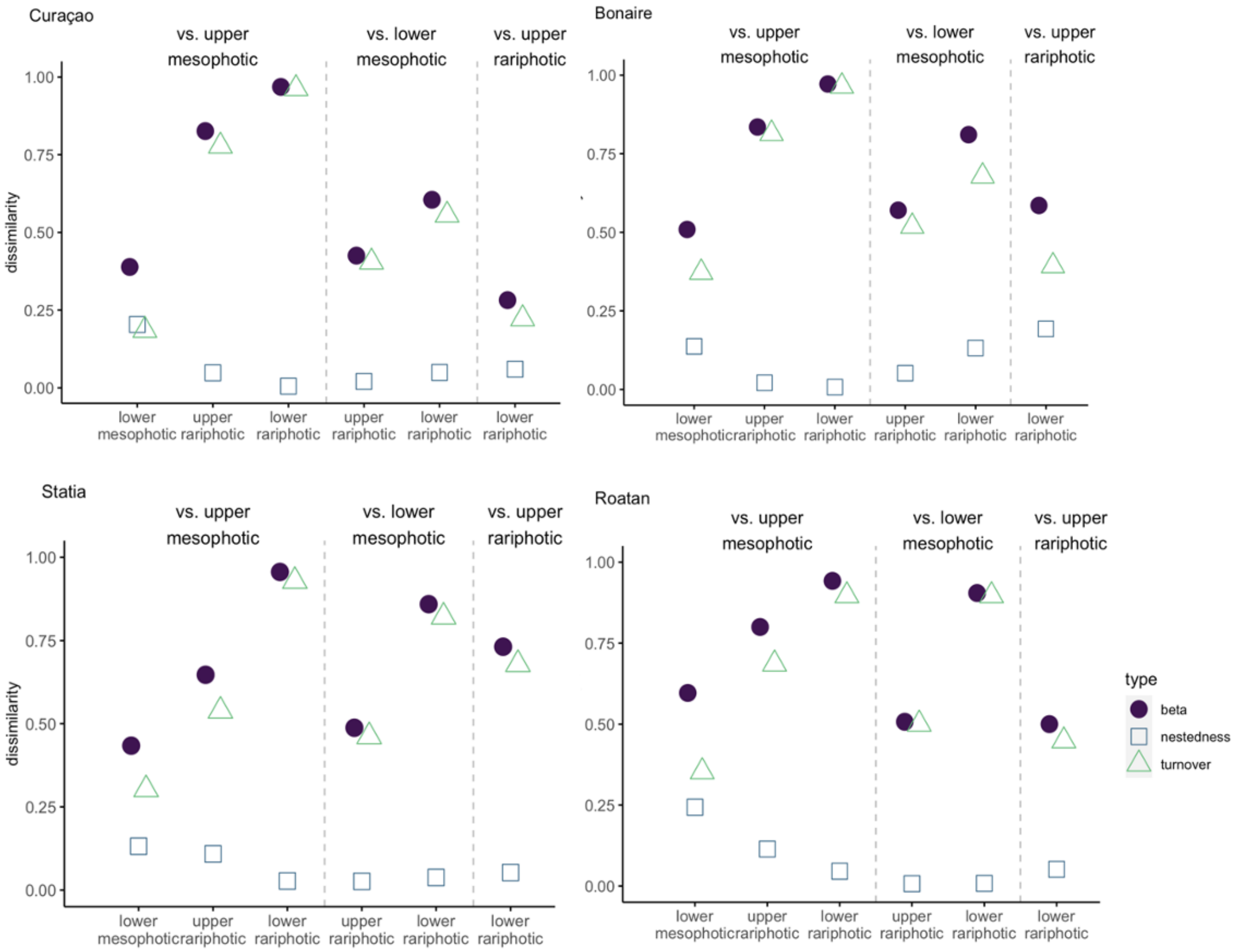

Figure S3: Beta-diversity and its components, nestedness and turnover, between reef-fish communities of different depth zones at each study site. Shape and color of points indicate values of overall beta-diversity (purple circles), nestedness (blue square), and turnover (green triangle).

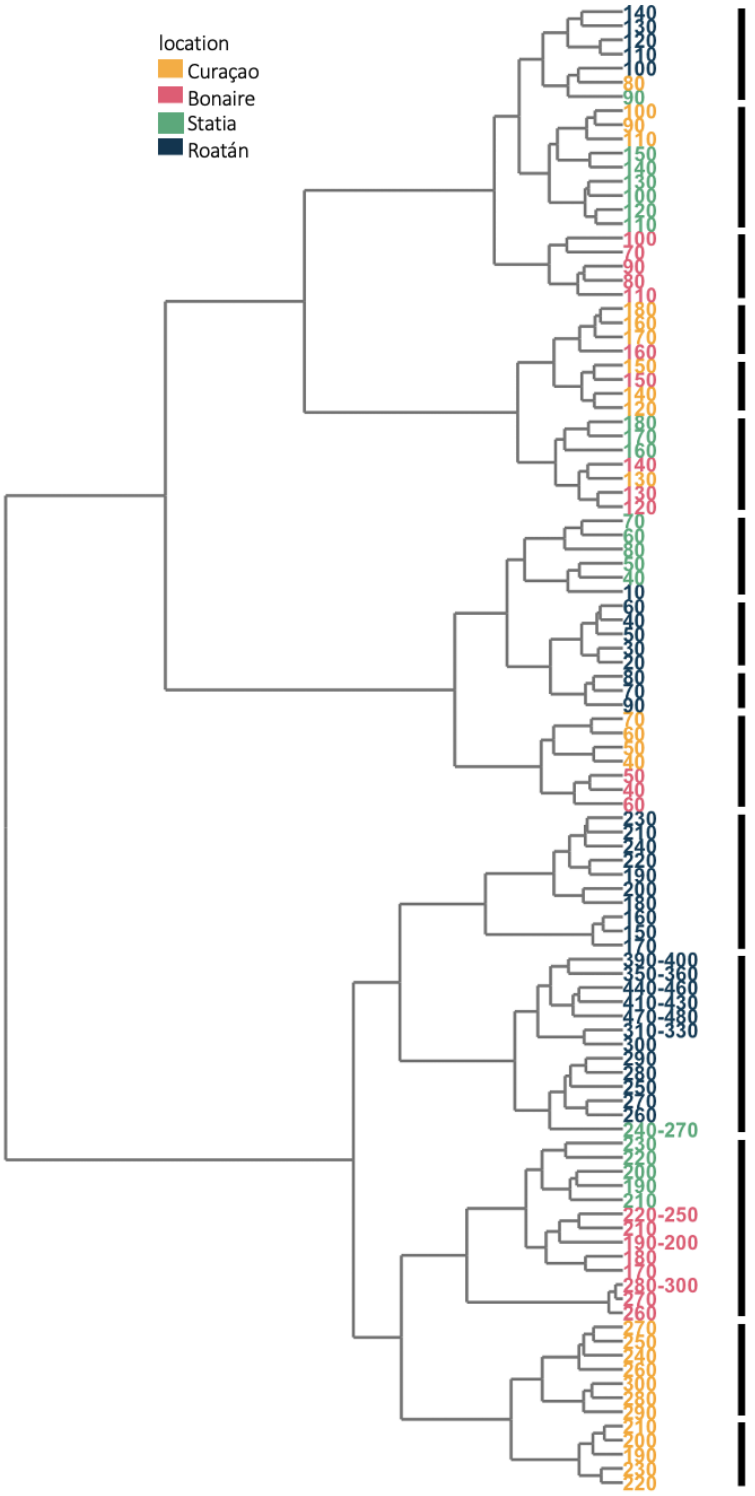

Figure S4: Trans-site hierarchical clustering analysis of deep-reef fish communities from 10 to 480 m. Length of branches in the dendrogram is commensurate to the dissimilarity between depth bins based on Bray-Curtis distance. Font color indicates sites. Significant clusters (SIMPROF analyses, Ward linkage) are indicated by thick black vertical lines.

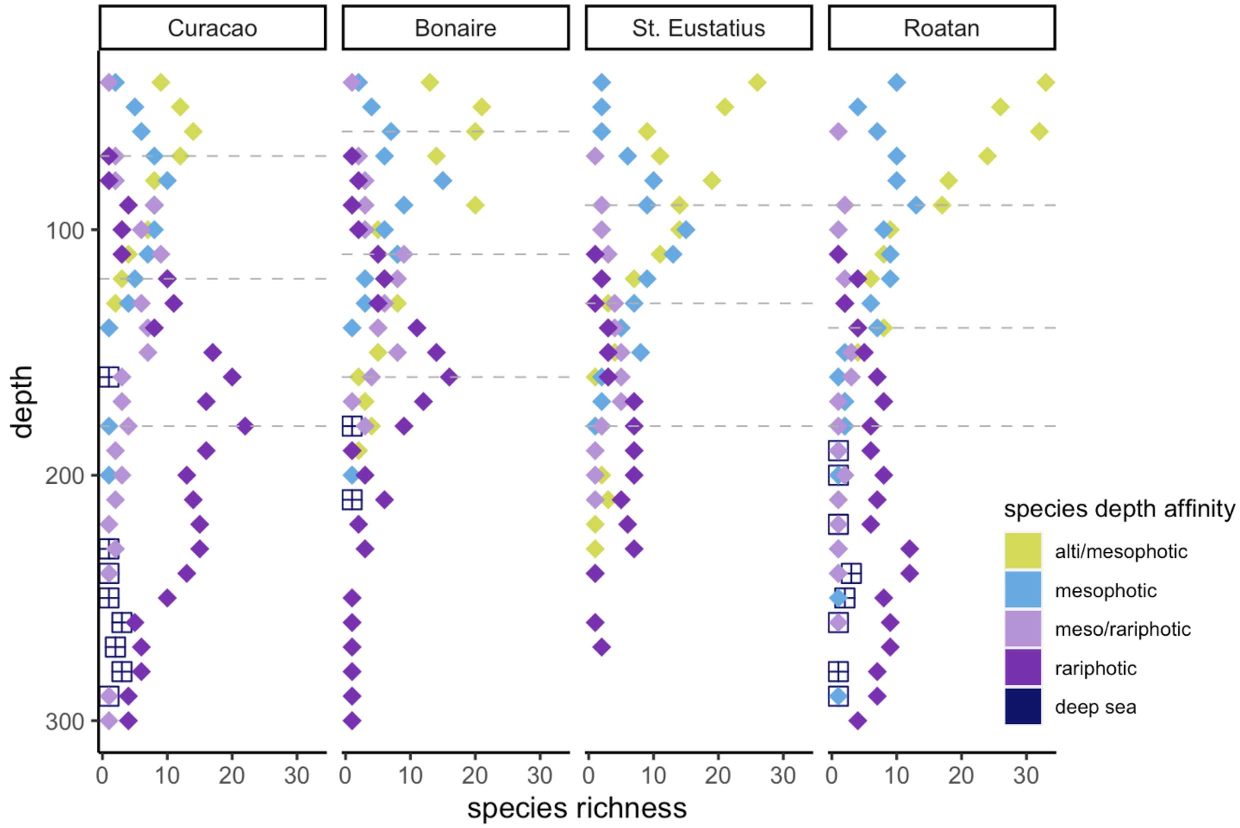

Figure S5: Contribution of depth affinity groups to fish richness across depth at the four study locations. Colors and symbols indicate depth affinities. Dashed lines represent the depth of site-specific community breaks.

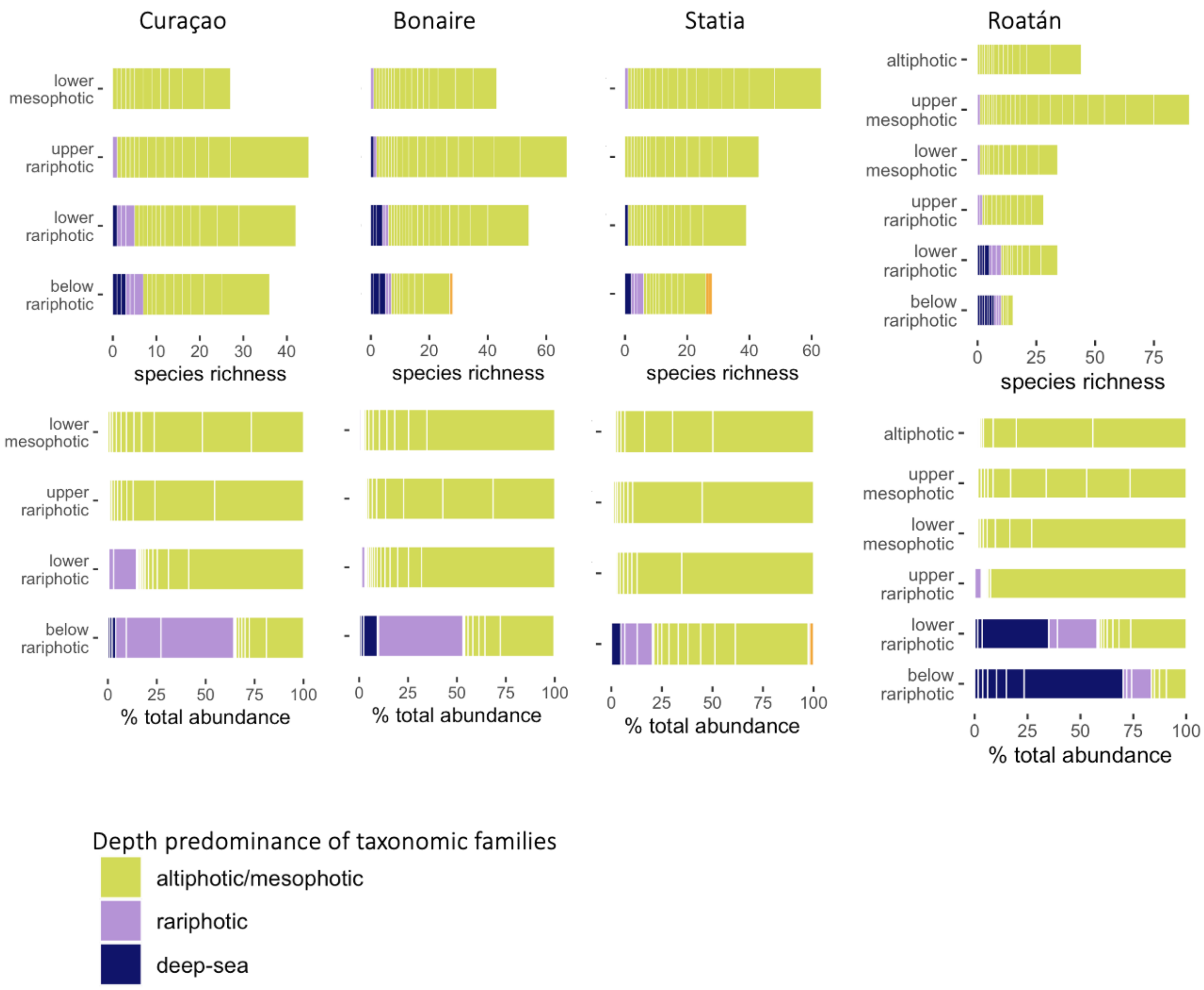

Figure S6: Depth-zone predominance of fish families in each depth zone and at each site and their contribution to species richness (top panels) and abundance (bottom panels). White lines in barplots distinguish species from different families. Filling colors indicate the depth-zone predominance of taxonomic families.

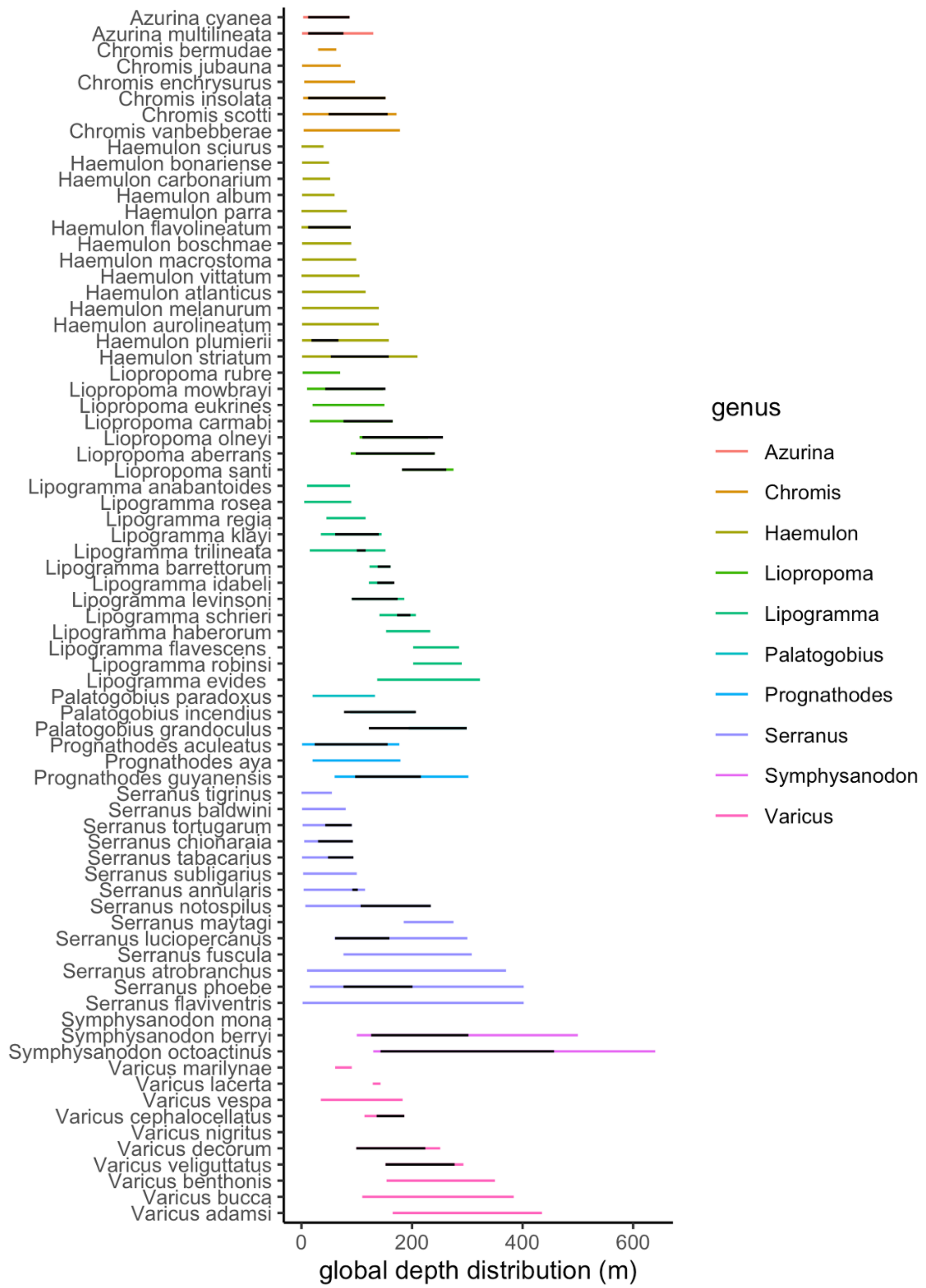

Figure S7: Depth range of the ten genera displayed in Figure 7. Colored lines represent global depth ranges (global minima and maxima), and black lines represent depth ranges observed at the sampling sites of this study. Colors denote distinct genera.

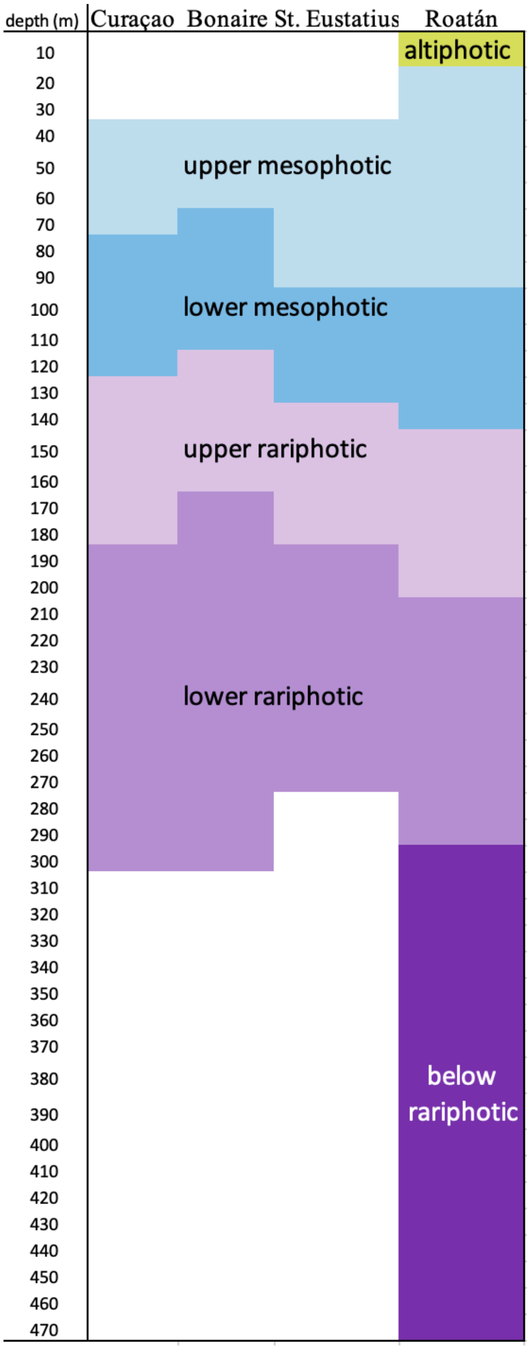

Figure S8: Site-specific limits of depth zones, as informed from fish-community dissimilarity analyses.

Table S1: List of species observed organized by taxonomic family. The predominant depth of families, the predominant depth of species, the presence (1) / absence (0) of the species at the four sites studied, and the overall minimal and maximal depths at which these species have been observed in the Greater Caribbean (Robertson and Van Tassell, 2023). The predominant depth of species are coded as follows: altiphotic/mesophotic (AM), mesophotic (M), mesophotic/rariphotic (MR), rariphotic (R), deep sea (DS). Species new to science at the time of their observation are indicated in bold font.

species	family predominant depth	species predominant depth	Curaçao	Bonaire	Statia	Roatán	overall min depth	overall max depth
**ACANTHURIDAE**								
Acanthurus chirurgus	altiphotic/mesophotic	AM	0	0	1	1	0	107
Acanthurus coeruleus	altiphotic/mesophotic	AM	0	1	1	1	0	76
Acanthurus tractus	altiphotic/mesophotic	AM	0	1	1	1	0	117
**ACROPOMATIDAE**								
Synagrops bellus	deep-sea	DS	0	1	0	1	60	1174
Synagrops egretta	deep-sea	DS	0	1	0	0	NA	NA
Verilus sordidus	deep-sea	MR	0	0	0	1	100	600
**ANTIGONIIDAE**								
Antigonia capros	rariphotic	R	1	0	1	0	27	900
**APOGONIDAE**								
Apogon gouldi	altiphotic/mesophotic	MR	1	1	0	1	55	262
Apogon lachneri	altiphotic/mesophotic	AM	0	0	0	1	5	106
Apogon maculatus	altiphotic/mesophotic	AM	0	1	0	0	1	128
Apogon pillionatus	altiphotic/mesophotic	AM	0	0	1	0	7	122
Apogon pseudomaculatus	altiphotic/mesophotic	AM	0	1	0	0	1	134
Paroncheilus affinis	altiphotic/mesophotic	M	1	1	1	0	15	300

**AULOPIDAE**								
Aulopus filamentosus	rariphotic	MR	0	0	0	1	50	1000
**AULOSTOMIDAE**								
Aulostomus maculatus	altiphotic/mesophotic	AM	0	0	0	1	2	300
**BALISTIDAE**								
Canthidermis sufflamen	altiphotic/mesophotic	AM	0	0	1	1	0.5	300
Melichthys niger	altiphotic/mesophotic	AM	0	0	0	1	0	406
Xanthichthys ringens	altiphotic/mesophotic	M	1	1	1	0	0	190
**BEMBROPIDAE**								
Bembrops sp.	rariphotic	R	0	0	0	1	NA	NA
Chrionema squamentum	rariphotic	R	1	1	1	1	115	306
**CALLIONYMIDAE**								
Foetorepus agassizii	altiphotic/mesophotic	R	1	0	0	0	NA	NA
**CARANGIDAE**								
Caranx bartholomaei	altiphotic/mesophotic	AM	0	1	0	0	0	104
Caranx ruber	altiphotic/mesophotic	AM	0	1	1	1	0	155
Caranx latus	altiphotic/mesophotic	AM	0	1	0	1	0	151
Caranx lugubris	altiphotic/mesophotic	M	0	0	1	1	3	380
Decapterus sp.	altiphotic/mesophotic	AM	0	0	0	1	NA	NA
Seriola dumerili	altiphotic/mesophotic	R	0	0	0	1	3	385
Seriola rivoliana	altiphotic/mesophotic	MR	0	0	1	0	3	340
Trachinotus goodei	altiphotic/mesophotic	M	0	0	0	1	0	40
**CHAETODONTIDAE**								
Chaetodon capistratus	altiphotic/mesophotic	AM	1	0	1	1	0	130

Chaetodon ocellatus	altiphotic/mesophotic	AM	0	0	0	1	0	110
Chaetodon sedentarius	altiphotic/mesophotic	AM	0	1	1	1	0	172
Chaetodon striatus	altiphotic/mesophotic	AM	0	1	1	1	0	151
Prognathodes aculeatus	altiphotic/mesophotic	M	1	1	1	1	1	177
Prognathodes guyanensis	altiphotic/mesophotic	MR	1	1	1	1	60	302
**CHAUNACIDAE**								
Chaunax pictus	deep-sea	DS	1	0	0	1	200	1183
**CYCLOPSETTIDAE**								
Syacium gunteri	generalist	R	0	1	0	0	10	174
**EPIGONIDAE**								
Epigonidae sp.	deep-sea	MR	0	0	0	1	NA	NA
Sphyraenops bairdianus	deep-sea	DS	1	0	0	0	200	1750
**FISTULARIIDAE**								
Fistularia sp.	altiphotic/mesophotic	AM	0	1	1	0	NA	NA
**GEMPYLIDAE**								
Neoepinnula americana	deep-sea	DS	0	0	0	1	166	1027
**GOBIESOCIDAE**								
Derilissus lombardii	altiphotic/mesophotic	M	0	0	1	0	30	132
**GOBIIDAE**								
Antilligobius nikkiae	**altiphotic/mesophotic**	**M**	**1**	**1**	**1**	**1**	**73**	**205**
Bollmannia boqueronensis	altiphotic/mesophotic	M	0	1	0	0	14	112
Bollmannia eigenmanni	altiphotic/mesophotic	M	0	1	0	0	37	200
Coryphopterus curasub	**altiphotic/mesophotic**	**M**	**1**	**1**	**0**	**0**	**70**	**97**
Coryphopterus personatus	altiphotic/mesophotic	AM	0	0	0	1	2	73

Elacatinus chancei	altiphotic/mesophotic	AM	0	0	1	1	4	119
Elacatinus lobeli	altiphotic/mesophotic	AM	0	0	0	1	2	27
Gnatholepis thompsoni	altiphotic/mesophotic	AM	0	1	1	1	0	85
Oxyurichthys stigmalophius	altiphotic/mesophotic	M	0	1	0	0	2	101
Palatogobius grandoculus	altiphotic/mesophotic	R	0	0	1	1	194	299
Palatogobius incendius	**altiphotic/mesophotic**	**MR**	**1**	**1**	**1**	**1**	**88**	**205**
Palatogobius paradoxus	altiphotic/mesophotic	R	0	0	0	1	20	133
Pinnichthys aimoriensis	altiphotic/mesophotic	MR	0	1	1	0	70	163
Priolepis hipoliti	altiphotic/mesophotic	AM	0	1	0	0	2	117
Psilotris laurae	**altiphotic/mesophotic**	**R**	**0**	**1**	**0**	**0**	**114**	**250**
Ptereleotris helenae	altiphotic/mesophotic	AM	1	1	1	1	3	180
Varicus adamsi	**altiphotic/mesophotic**	**R**	**0**	**0**	**0**	**1**	**165**	**435**
Varicus cephalocellatus	**altiphotic/mesophotic**	**R**	**0**	**1**	**1**	**0**	**114**	**186**
Varicus decorum	**altiphotic/mesophotic**	**R**	**1**	**1**	**0**	**0**	**99**	**251**
Varicus veliguttatus	**altiphotic/mesophotic**	**R**	**1**	**0**	**1**	**1**	**152**	**293**
Vomerogobius flavus	altiphotic/mesophotic	M	0	0	0	1	14	42
**GRAMMATIDAE**								
Gramma linki	altiphotic/mesophotic	M	0	0	1	1	20	180
Gramma loreto	altiphotic/mesophotic	AM	1	1	0	1	2	130
Gramma melacara	altiphotic/mesophotic	M	0	0	0	1	15	180
Lipogramma barrettorum	**altiphotic/mesophotic**	**R**	**1**	**0**	**0**	**0**	**NA**	**NA**
Lipogramma evides	altiphotic/mesophotic	R	1	1	1	1	NA	NA
Lipogramma flavescens	**altiphotic/mesophotic**	**MR**	**0**	**0**	**0**	**1**	**NA**	**NA**
Lipogramma haberi	**altiphotic/mesophotic**	**R**	**1**	**0**	**0**	**0**	**NA**	**NA**

Lipogramma idabeli	**altiphotic/mesophotic**	**MR**	**0**	**0**	**0**	**1**	**122**	**165**
Lipogramma klayi	altiphotic/mesophotic	M	1	1	1	1	NA	NA
Lipogramma levinsoni	**altiphotic/mesophotic**	**MR**	**1**	**1**	**1**	**1**	**NA**	**NA**
Lipogramma regium	altiphotic/mesophotic	M	0	0	1	0	NA	NA
Lipogramma schrieri	**altiphotic/mesophotic**	**R**	**1**	**0**	**0**	**0**	**NA**	**NA**
Lipogramma trilineata	altiphotic/mesophotic	M	0	0	1	0	NA	NA
**HAEMULIDAE**								
Anisotremus surinamensis	altiphotic/mesophotic	AM	0	1	0	0	0	109
Anisotremus virginicus	altiphotic/mesophotic	AM	0	0	0	1	1	112
Haemulon bonariense	altiphotic/mesophotic	AM	0	0	0	1	1	50
Haemulon chrysargyreum	altiphotic/mesophotic	AM	0	1	0	0	0	42
Haemulon flavolineatum	altiphotic/mesophotic	AM	1	1	0	1	0	89
Haemulon plumierii	altiphotic/mesophotic	AM	0	0	0	1	1	158
Haemulon sciurus	altiphotic/mesophotic	AM	0	0	0	1	0	40
Haemulon striatum	altiphotic/mesophotic	AM	1	1	1	1	1	210
Haemulon vittata	altiphotic/mesophotic	AM	1	0	0	0	0	105
**HOLOCENTRIDAE**								
Corniger spinosus	altiphotic/mesophotic	R	1	1	1	1	42	275
Flammeo marianus	altiphotic/mesophotic	M	1	1	1	1	15	151
Holocentrus adscensionis	altiphotic/mesophotic	AM	0	1	1	0	1	274
Holocentrus rufus	altiphotic/mesophotic	AM	0	0	1	1	1	330
Myripristis jacobus	altiphotic/mesophotic	AM	1	1	1	0	1	210
Neoniphon coruscum	altiphotic/mesophotic	AM	0	1	0	0	2	140
Ostichthys trachypoma	altiphotic/mesophotic	R	1	1	1	1	37	550

Sargocentron bullisi	altiphotic/mesophotic	MR	0	1	0	0	0	128
Sargocentron poco	altiphotic/mesophotic	AM	0	0	0	1	2	152
**LABRIDAE**								
Bodianus pulchellus	altiphotic/mesophotic	AM	0	0	0	1	10	120
Bodianus rufus	altiphotic/mesophotic	AM	0	1	1	1	1	130
Clepticus parrae	altiphotic/mesophotic	AM	1	1	1	1	0	145
Decodon puellaris	altiphotic/mesophotic	R	1	0	1	0	18	275
Decodon shallow	**altiphotic/mesophotic**	**MR**	**1**	**1**	**1**	**0**	**NA**	**NA**
Halichoeres bathyphilus	altiphotic/mesophotic	M	0	1	1	0	18	275
Halichoeres garnoti	altiphotic/mesophotic	AM	0	1	1	1	2	130
Halichoeres maculipinna	altiphotic/mesophotic	AM	0	0	0	1	2	50
Halichoeres pictus	altiphotic/mesophotic	AM	0	0	0	1	5	55
Lachnolaimus maximus	altiphotic/mesophotic	AM	0	0	0	1	0	91
Polylepion sp.	altiphotic/mesophotic	MR	1	0	0	1	NA	NA
Scarus iseri	altiphotic/mesophotic	AM	0	0	0	1	0	68
Scarus taeniopterus	altiphotic/mesophotic	AM	0	1	1	1	1	107
Scarus vetula	altiphotic/mesophotic	AM	0	1	0	1	1	71
Sparisoma atomarium	altiphotic/mesophotic	AM	0	0	0	1	0	106
Sparisoma aurofrenatum	altiphotic/mesophotic	AM	0	0	1	1	1	117
Sparisoma chrysopterum	altiphotic/mesophotic	AM	0	0	0	1	1	61
Sparisoma rubripinne	altiphotic/mesophotic	AM	0	0	0	1	0	60
Sparisoma viride	altiphotic/mesophotic	AM	0	1	1	1	0	107
Thalassoma bifasciatum	altiphotic/mesophotic	AM	0	0	1	1	0	130
**LABRISOMIDAE**								

Haptoclinus dropi	**altiphotic/mesophotic**	**MR**	**1**	**0**	**0**	**1**	**157**	**274**
**LATILIDAE**								
Caulolatilus dooleyi	deep-sea	AM	0	1	0	0	208	256
**LUTJANIDAE**								
Apsilus dentatus	altiphotic/mesophotic	M	0	0	0	1	12	300
Etelis oculatus	altiphotic/mesophotic	R	0	0	0	1	100	533
Ocyurus chrysurus	altiphotic/mesophotic	AM	0	1	0	1	0	180
Pristipomoides aquilonaris	altiphotic/mesophotic	R	0	1	0	0	24	650
Pristipomoides freemani	altiphotic/mesophotic	R	0	1	0	0	61	220
Pristipomoides macrophthalmus	altiphotic/mesophotic	R	1	0	1	0	100	611
**MALACANTHIDAE**								
Malacanthus plumieri	rariphotic	AM	0	1	1	1	10	184
**MONACANTHIDAE**								
Cantherhines sp.	altiphotic/mesophotic	AM	0	0	0	1	NA	NA
**MORIDAE**								
Physiculus fulvus	deep-sea	R	0	1	0	0	69	800
**MULLIDAE**								
Mulloidichthys martinicus	altiphotic/mesophotic	AM	0	1	1	1	1	135
Pseudupeneus maculatus	altiphotic/mesophotic	AM	0	0	0	1	1	110
**MURAENIDAE**								
Gymnothorax funebris	altiphotic/mesophotic	AM	0	1	0	0	0	286
Gymnothorax moringa	altiphotic/mesophotic	AM	0	1	0	0	0	304
Gymnothorax ocellatus	altiphotic/mesophotic	AM	0	1	1	0	1	445
Muraena retifera	altiphotic/mesophotic	AM	0	1	0	0	20	169

**BATHYCLUPEIDAE**								
Bathyclupea sp.	deep-sea	DS	0	0	0	1	NA	NA
**OGCOCEPHALIDAE**								
Ogcocephalus corniger	deep-sea	R	0	0	1	0	29	253
Ogcocephalus parvus	deep-sea	R	0	1	0	0	29	360
Zalieutes mcgintyi	deep-sea	R	0	0	1	0	90	660
**OPHIDIIDAE**								
Brotula barbata	generalist	R	0	0	1	0	1	650
**OPISTOGNATHIDAE**								
Opistognathus aurifrons	altiphotic/mesophotic	AM	0	0	0	1	2	65
Opistognathus schrieri	**altiphotic/mesophotic**	**R**	**1**	**0**	**0**	**1**	**152**	**152**
**OSTRACIIDAE**								
Acanthostracion polygonius	altiphotic/mesophotic	AM	0	0	1	0	2	121
Acanthostracion quadricornis	altiphotic/mesophotic	AM	0	0	1	1	2	108
Lactophrys trigonus	altiphotic/mesophotic	AM	0	0	0	1	1	110
Lactophrys triqueter	altiphotic/mesophotic	AM	0	1	0	1	2	79
**PARALICHTHYIDAE**								
Gastropsetta frontalis	generalist	AM	0	0	1	0	35	212
**POMACANTHIDAE**								
Centropyge argi	altiphotic/mesophotic	AM	1	1	1	1	3	170
Holacanthus ciliaris	altiphotic/mesophotic	AM	0	1	1	1	1	128
Holacanthus tricolor	altiphotic/mesophotic	AM	0	0	1	1	1	143
Pomacanthus arcuatus	altiphotic/mesophotic	AM	0	1	0	1	1	101
Pomacanthus paru	altiphotic/mesophotic	AM	0	1	1	1	1	157

Abudefduf saxatilis	altiphotic/mesophotic	AM	0	0	0	1	0	41
Azurina cyanea	rariphotic	AM	1	1	1	1	3	87
Chromis vanbebberae	**altiphotic/mesophotic**	**MR**	**1**	**1**	**1**	**1**	**4**	**178**
Chromis insolata	altiphotic/mesophotic	AM	1	1	1	1	3	152
Azurina multilineata	altiphotic/mesophotic	AM	1	1	1	1	1	130
Chromis scotti	altiphotic/mesophotic	M	1	1	1	1	2	172
Microspathodon chrysurus	altiphotic/mesophotic	AM	0	0	0	1	0	62
Stegastes diencaeus	altiphotic/mesophotic	AM	0	0	0	1	0	45
Stegastes partitus	altiphotic/mesophotic	AM	1	1	1	1	0	130
Stegastes planifrons	altiphotic/mesophotic	AM	0	0	0	1	0	43
Stegastes variabilis	altiphotic/mesophotic	AM	0	0	0	1	NA	NA
Stegastes xanthurus	altiphotic/mesophotic	MR	0	0	0	1	0	100
**PRIACANTHIDAE**								
Heteropriacanthus cruentatus	altiphotic/mesophotic	R	0	0	0	1	5	432
Priacanthus arenatus	altiphotic/mesophotic	R	0	0	1	1	10	587
Pristigenys alta	altiphotic/mesophotic	R	1	1	1	1	5	300
**SCIAENIDAE**								
Equetus lanceolatus	altiphotic/mesophotic	AM	0	1	1	0	2	230
**SCORPAENIDAE**								
Phenacoscorpius nebris	altiphotic/mesophotic	MR	0	0	0	1	64	606
Pontinus castor	altiphotic/mesophotic	R	1	1	1	1	32	549
Pontinus nematophthalmus	altiphotic/mesophotic	R	1	0	0	0	82	698
Pterois volitans	altiphotic/mesophotic	AM	0	0	0	1	2	304
Scorpaena brasiliensis	altiphotic/mesophotic	R	0	1	0	0	1	204

Scorpaenodes barrybrowni	**altiphotic/mesophotic**	**R**	**0**	**1**	**0**	**0**	**95**	**235**
Scorpaenodes caribbaeus	altiphotic/mesophotic	R	0	0	1	0	0	70
**SERRANIDAE**								
Anthias asperilinguis	altiphotic/mesophotic	MR	1	0	0	1	69	393
Baldwinella vivanus	**altiphotic/mesophotic**	**R**	**1**	**1**	**1**	**0**	**20**	**610**
Bathyanthias sp.	**altiphotic/mesophotic**	**R**	**0**	**1**	**1**	**0**	**NA**	**NA**
Bullisichthys caribbaeus	altiphotic/mesophotic	M	1	1	1	1	81	548
Centropristis fuscula	altiphotic/mesophotic	MR	1	0	1	0	NA	NA
Cephalopholis cruentata	altiphotic/mesophotic	AM	1	1	1	1	1	170
Cephalopholis fulva	altiphotic/mesophotic	AM	0	0	1	1	1	350
Choranthias tenuis	altiphotic/mesophotic	MR	1	1	0	0	55	915
Epinephelus striatus	altiphotic/mesophotic	AM	0	0	0	1	5	255
Epinephelus adscensionis	altiphotic/mesophotic	AM	0	0	1	0	2	189
Epinephelus guttatus	altiphotic/mesophotic	AM	0	0	1	0	2	181
Gonioplectrus hispanus	altiphotic/mesophotic	R	1	1	1	1	35	460
Hemanthias leptus	altiphotic/mesophotic	MR	1	1	0	0	35	640
Hypoplectrus chlorurus	altiphotic/mesophotic	AM	0	1	0	0	3	74
Hypoplectrus indigo	altiphotic/mesophotic	AM	0	0	1	1	3	54
Hypoplectrus nigricans	altiphotic/mesophotic	AM	0	0	0	1	1	67
Hypoplectrus puella	altiphotic/mesophotic	AM	0	0	1	1	3	90
Hyporthodus niveatus	altiphotic/mesophotic	R	0	1	0	0	10	1066
Jeboehlkia gladifer	altiphotic/mesophotic	R	1	1	0	1	100	395
Liopropoma aberrans	altiphotic/mesophotic	R	1	1	0	1	89	241
Liopropoma carmabi	altiphotic/mesophotic	M	0	1	1	0	15	165

Liopropoma mowbrayi	altiphotic/mesophotic	M	1	1	1	1	10	148
Liopropoma olneyi	**altiphotic/mesophotic**	**R**	**1**	**1**	**1**	**1**	**105**	**229**
Liopropoma santi	**altiphotic/mesophotic**	**R**	**1**	**0**	**0**	**1**	**182**	**275**
Mycteroperca bonaci	altiphotic/mesophotic	M	0	1	0	1	1	250
Mycteroperca interstitialis	altiphotic/mesophotic	M	0	1	0	1	2	150
Mycteroperca tigris	altiphotic/mesophotic	AM	0	0	0	1	3	135
Paranthias furcifer	altiphotic/mesophotic	AM	1	1	1	0	0	128
Plectranthias garrupellus	altiphotic/mesophotic	R	0	0	1	1	13	375
Plectranthias sp.	**altiphotic/mesophotic**	**MR**	**1**	**1**	**1**	**0**	**NA**	**NA**
Pronotogrammus martinicensis	altiphotic/mesophotic	MR	1	1	1	1	35	900
Rypticus maculatus	altiphotic/mesophotic	AM	0	1	0	0	3	100
Rypticus saponaceus	altiphotic/mesophotic	AM	0	1	1	1	0	213
Serranus annularis	altiphotic/mesophotic	AM	0	0	1	0	4	115
Serranus atrobranchus	altiphotic/mesophotic	R	0	1	0	0	10	370
Serranus chionaraia	altiphotic/mesophotic	AM	0	1	0	1	5	93
Serranus luciopercanus	altiphotic/mesophotic	M	1	1	1	1	60	300
Serranus notospilus	altiphotic/mesophotic	R	1	1	1	0	7	234
Serranus phoebe	altiphotic/mesophotic	R	1	1	1	0	15	402
Serranus tabacarius	altiphotic/mesophotic	AM	0	1	1	1	1	94
Serranus tigrinus	altiphotic/mesophotic	M	0	1	0	0	0	55
Serranus tortugarum	altiphotic/mesophotic	M	0	1	1	1	2	91
**SPARIDAE**								
Calamus calamus	altiphotic/mesophotic	AM	0	0	0	1	1	84
**SPHYRAENIDAE**								

Sphyraena barracuda	altiphotic/mesophotic	AM	0	1	1	1	0	450
**SYMPHYSANODONTIDAE**								
Symphysanodon berryi	rariphotic	R	1	0	1	1	100	500
Symphysanodon octoactinus	rariphotic	R	1	1	1	1	130	640
**TETRAODONTIDAE**								
Canthigaster jamestyleri	altiphotic/mesophotic	AM	1	1	0	1	14	218
Canthigaster rostrata	altiphotic/mesophotic	AM	0	1	1	1	1	155
Sphoeroides dorsalis	altiphotic/mesophotic	AM	0	0	1	0	8	324
**TRACHICHTHYIDAE**								
Gephyroberyx darwinii	deep-sea	DS	1	0	0	1	10	1250
**TRIACANTHODIDAE**								
Hollardia hollardi	deep-sea	DS	0	0	0	1	230	915
**TRIGLIDAE**								
Bellator brachychir	altiphotic/mesophotic	R	1	1	0	0	27	366
Bellator egretta	altiphotic/mesophotic	R	1	1	1	0	40	232
Peristedion brevirostre	altiphotic/mesophotic	R	0	1	0	0	55	550

Table S3. Species significantly contributing to the differences between depth zones at each site (SIMPER analyses). Only species that contributed to >5% of dissimilarity, with p-value<0.05 are shown.

		upper vs. lower mesophotic		lower mesophotic vs. upper rariphotic		upper vs. lower rariphotic	
	**species**	**contribution**	**p-value**	**contribution**	**p-value**	**contribution**	**p-value**
Bonaire	*Serranus phoebe*					0.052	0.002
	Pronotogrammus martinicensis					0.137	0.001
Curaçao	*Pronotogrammus martinicensis*					0.076	0.001
	Clepticus parrae	0.060	0.009				
Roatan	*Symphysanodon octoactinus*					0.052	0.001
	Palatogobius grandoculus					0.131	0.001
Statia	*Palatogobius incendius*					0.200	0.001
	Canthigaster rostrata			0.063	0.028		
	Clepticus parrae	0.054	0.004				
	Chromis insolata	0.092	0.003				
	Pronotogrammus martinicensis					0.053	0.003
	Palatogobius incendius					0.097	0.002
	Antilligobius nikkiae					0.131	0.004
	Bullisichthys caribbaeus			0.096	0.018		

Table S4: Kruskal-Wallis and Wilcoxon rank sum test assessing differences in the depth distribution of species within the same genera (depth~species).

	Kruskal-Wallis and Wilcoxon rank sum test		
	chi-squared	df	p-value
Lipogramma	241.5	2	**<0.001**
klayi - evides			**<0.001**
levinsoni - evides			**<0.001**
levinsoni - klayi			**<0.001**
Liopropoma	285.3	2	**<0.001**
aberrans-olneyi			**0.04**
mowbrayi-olneyi			**<0.001**
mowbrayi-aberrans			**<0.001**
Serranus	436.2	3	**<0.001**
phoebe-notospilus			**<0.001**
luciopercanus-notospilus			**<0.001**
tortugarum-notospilus			**<0.001**
luciopercanus-phoebe			**<0.001**
tortugarum-phoebe			**<0.001**
tortugarum-luciopercanus			**<0.001**

Table S5: Shapiro test of normality and Wilcoxon rank sum test (depth~species) for genera with two species evaluated.

	**Shapiro test**		**Wilcoxon rank sum test**	
	W	p-value	W	p-value
Azurina	0.83	<0.001	200158.00	**<0.001**
Chromis	0.95	<0.001	1096354.00	**<0.001**
Prognathodes	0.99	0.15	4873.00	**<0.001**
Palatogobius	0.92	<0.001	2688437.00	**<0.001**
Symphysonadon	0.94	<0.001	40802.00	**<0.001**
Haemulon	0.9	<0.001	11895.00	**<0.001**
Varicus	0.94	0.11	177.00	**<0.001**
Liopropoma	0.98	<0.001		
Lipogramma	0.87	<0.001		
Serranus	0.96	<0.001		

Table S6: Species observed below the rariphotic (300 - 480 m) in Roatán and their abundance before (raw) and after (normalized) correcting for sampling effort.

species	abundance normalized	raw abundance
Epigonidae sp.	162.059641	25
Hollardia hollardi	29.141401	7
Gephyroberyx darwinii	14.462922	4
Chrionema squamentum	32.363636	4
Jeboehlkia gladifer	24.272727	3
Neoepinnula americana	7.242157	2
Phenacoscorpius nebris	11.269481	2
Chaunax pictus	16.181818	2
Polylepion sp.	5.235294	1
Neoscopelus macrolepidotus	5.933333	1
Symphysanodon octoactinus	5.933333	1
Anthias asperilinguis	8.090909	1
Aulopus filamentosus	8.090909	1
Haptoclinus dropi	8.090909	1
Verilus sordidus	8.090909	1

Table S7: Summary statistics of the PERMANOVA testing for the effect of depth zone, site location, and the interaction between these two factors, on fish community structure.

	**Df**	**Sum of squares**	**R2**	**F**	**Pr(>F)**
depth zone	1	6.45	0.16	23.31	**0.001**
location	3	5.20	0.13	6.26	**0.001**
depth zone : location	3	3.57	0.08	4.30	**0.001**
Residual	95	26.27	0.63		
Total	102	41.49	1		
